# Reduced interleukin-18 secretion by human monocytic cells in response to infections with hyper-virulent *Streptococcus pyogenes*

**DOI:** 10.1186/s12929-024-01014-9

**Published:** 2024-02-27

**Authors:** Lea A. Tölken, Antje D. Paulikat, Lana H. Jachmann, Alexander Reder, Manuela Gesell Salazar, Laura M. Palma Medina, Stephan Michalik, Uwe Völker, Mattias Svensson, Anna Norrby-Teglund, Katharina J. Hoff, Michael Lammers, Nikolai Siemens

**Affiliations:** 1 Department of Molecular Genetics and Infection Biology, University of Greifswald, Greifswald, Germany; 2 Department of Functional Genomics, University Medicine Greifswald, Greifswald, Germany; 3 Center for Infectious Medicine, Karolinska Institutet, Karolinska University Hospital, Huddinge, Sweden; 4 Institute of Mathematics and Computer Science, University of Greifswald, Greifswald, Germany; 5 Department of Synthetic and Structural Biochemistry, Institute of Biochemistry, University of Greifswald, Greifswald, Germany

**Keywords:** *Streptococcus pyogenes*, Dendritic cells, Interleukin-18, CovR/S, Necrotizing soft tissue infection

## Abstract

**Background:**

*Streptococcus pyogenes* (group A streptococcus, GAS) causes a variety of diseases ranging from mild superficial infections of the throat and skin to severe invasive infections, such as necrotizing soft tissue infections (NSTIs). Tissue passage of GAS often results in mutations within the genes encoding for control of virulence (Cov)R/S two component system leading to a hyper-virulent phenotype. Dendritic cells (DCs) are innate immune sentinels specialized in antigen uptake and subsequent T cell priming. This study aimed to analyze cytokine release by DCs and other cells of monocytic origin in response to wild-type and natural *covR/S* mutant infections.

**Methods:**

Human primary monocyte-derived (mo)DCs were used. DC maturation and release of pro-inflammatory cytokines in response to infections with wild-type and *covR/S* mutants were assessed via flow cytometry. Global proteome changes were assessed via mass spectrometry. As a proof-of-principle, cytokine release by human primary monocytes and macrophages was determined.

**Results:**

In vitro infections of moDCs and other monocytic cells with natural GAS *covR/S* mutants resulted in reduced secretion of IL-8 and IL-18 as compared to wild-type infections. In contrast, moDC maturation remained unaffected. Inhibition of caspase-8 restored secretion of both molecules. Knock-out of streptolysin O in GAS strain with unaffected CovR/S even further elevated the IL-18 secretion by moDCs. Of 67 fully sequenced NSTI GAS isolates, 28 harbored mutations resulting in dysfunctional CovR/S. However, analyses of plasma IL-8 and IL-18 levels did not correlate with presence or absence of such mutations.

**Conclusions:**

Our data demonstrate that strains, which harbor *covR/S* mutations, interfere with IL-18 and IL-8 responses in monocytic cells by utilizing the caspase-8 axis. Future experiments aim to identify the underlying mechanism and consequences for NSTI patients.

**Supplementary Information:**

The online version contains supplementary material available at 10.1186/s12929-024-01014-9.

## Background

*Streptococcus pyogenes* (group A streptococcus, GAS) causes a variety of human diseases ranging from mild superficial to severe invasive infections, including necrotizing soft tissue infections (NSTIs). Globally, invasive GAS infections are estimated to cause approx. 500,000 deaths annually [13]. NSTIs encompass necrosis of any layer of the skin and soft tissue compartment and are often associated with systemic toxicity. Despite prompt antibiotic treatment, intensive care, and extensive surgical intervention [2], the morbidity and mortality of such infections remain substantial [41, 59]. While microbial etiologies of NSTIs are diverse, GAS are among the main causative pathogens of monomicrobial NSTIs [11, 41]. In a recent Scandinavian prospective multicenter study INFECT, 31% of surgically confirmed NSTI cases were caused by GAS [12, 41].

In the host, GAS can adopt a hyper-virulent phenotype, often mediated by inactivating mutations within genes encoding for the two component system (TCS) control of virulence (Cov)R/S [18, 21, 60, 61, 69]. Strains possessing *covR/S* mutations are frequently isolated from patients suffering from invasive GAS infections [5, 27, 41, 63]. In mouse studies, GAS hyper-virulence is characterized by induction of excessive inflammation through increased superantigenic activity [6]. However, immunosuppressive effects, including impaired neutrophil responses, are also reported [66]. The CovR/S system consists of the histidine kinase CovS and the response regulator CovR. CovS senses environmental signals including pH acidification [19, 26]. Upon activation, CovS phosphorylates CovR at D53. Phosphorylated CovR binds DNA and activates or represses transcription of several target genes [19, 26]. CovR/S regulates up to 15% of the GAS genome and is a transcriptional suppressor of several key virulence factors, including the hyaluronic acid capsule, M protein, and cholesterol-dependent cytolysin streptolysin O (SLO). Consequently, inactivating mutations in *covR/S* result in higher expression of these virulence factors, among others [15, 60].

Dendritic cells (DCs) are sentinel leukocytes and proficient antigen presenting cells (APCs). They bridge the innate and adaptive arms of the immune system by presenting processed antigens to T cells [57]. After ingestion of pathogens, DCs mature, migrate to the secondary lymph nodes, and present antigens to T cells via major histocompatibility complex (MHC) class II molecules [58]. In addition, DCs express co-stimulatory molecules and secrete cytokines to shape the direction of T cell priming and differentiation [43]. Only limited data exists on DC-GAS interactions. In murine models, it was shown that DCs are essential to limit GAS spread to the lymph nodes [40]. Furthermore, *in vitro* studies have demonstrated that GAS inhibit DC maturation in a capsule- and/or SLO-dependent manner and induce significant cell death [16, 36]. In addition, the streptococcal DNase Sda1 was found to impair plasmacytoid dendritic cell recruitment by reducing IFN-1 levels at the tissue site of infection [35].

DCs and other myeloid cells of monocytic lineage are major sources of cytokine/chemokine production for an appropriate response to infections. To resist immuno-clearance, GAS interfere with chemokine/cytokine response. Extracellular interleukin (IL-)8 is degraded via SpyCEP [53]. SpyCEP is a surface-exposed subtilisin-like serine protease, which preferably cleaves CXC chemokines. Furthermore, intracellularly located GAS disrupt the Golgi-related network in macrophages through NADase Nga, which results in inhibition of IL-8 secretion through a yet unknown mechanism [47]. In addition to IL-8, DCs secrete IL-1β and IL-18. Both IL-1-family cytokines are stored intracellularly as inactive precursors. Processing and release occur upon activation of the NLRP3 inflammasome. The associated active executioner caspase-1 cleaves the precursors into the respective mature cytokines. Subsequently, the release is mediated through caspase-1 processed gasdermin D pores [24]. The secreted GAS cysteine protease SpeB was shown to cleave pro-IL-1β and pro-IL-18 into their respective mature forms [29, 38]. Both cytokines have strong pro-inflammatory effects and play crucial roles in invasive GAS infections. IL-1β network was identified as a key network involved in susceptibility to GAS NSTIs [14]. IL-18 is crucial for activation of mucosa associated invariant T (MAIT) cells and subsequent interferon (IFN)-γ secretion [45, 71]. Excessive MAIT cell activation through IL-12/IL-18 was linked to pathological cytokine storm underlying STSS [20], which is a common complication of NSTIs [46, 48].

To date, data on GAS-DCs interactions are scarce and mainly derived from murine studies. Thus, we investigated the cytokine response of human monocyte-derived (mo)DCs and other monocytic cells to infections with either wild-type GAS or natural *covR/S* mutants. All cells of monocytic lineage infected with *covR/S* mutants secreted significantly less IL-8 and IL-18 as compared to wild-type infections. Inhibition of host initiator caspase-8 restored the secretion of both cytokines in response to infections. Validation of systemic plasma IL-18 levels in patients did not show differences based on bacterial CovR/S assignment.

## Methods

### Bacterial and strains

The following GAS strains were used: NSTI/STSS strains 5448, 5626, 8003, and 8157 (provided by Donald E. Low, Mount Sinai Hospital, Toronto, Canada) [28, 34], 2002 and 2006 from the INFECT cohort [54], MGAS5005 [60], 5448AP [69], 5448Δ*emm1* [39], and 5448Δ*slo* [64]. All strains were cultured in Todd-Hewitt broth (Carl Roth) supplemented with 1.5% (w/v) yeast extract (Carl Roth) at 37 °C. Hemolytic activity of GAS stationary culture supernatants was assessed as previously described [36]. To assess CovR/S functionality, SpeB secretion and activity were tested via casein digestion assay [54]. Serial dilutions of bacteria were plated on modified Columbia agar containing 3% (w/v) skim milk (both Sigma-Aldrich) following incubation under 37 °C and 5% CO_2_ atmosphere for 24 h. SpeB producers, which harbor functional CovR/S, were characterized by a clearance zone around the colonies, whereas non-producers (dysfunctional CovR/S) had no zone of clearance.

### Isolation of human monocytes and generation of dendritic cells and macrophages

Human monocytes were isolated from buffy coats using CD14 S-pluriBead antihuman beads (PluriSelect) or EasySep Human CD14 Positive Selection Kit II (Stemcell technologies) according to manufacturer’s instructions. The moDCs were generated by culturing monocytes in well plates for 5 d in RPMI1640 (Cytivia) medium supplemented with 10% (v/v) heat inactivated fetal calf serum (FCS; Thermo Fisher), 89 ng × mL^−1^ GM-CSF, and 22 ng × mL^−1^ IL-4 (both Immunotools). Medium was exchanged on day 3.

The monocyte-derived macrophages were generated by culturing monocytes in well plates in RPMI1640 (Cytivia) medium supplemented with 10% (v/v) heat inactivated FCS (Thermo Fisher) and 25 ng × mL^−1^ GM-CSF (Immunotools) for 7 d. Medium was exchanged on days 3 and 5.

### Infections of myeloid cells

All infections were performed in RPMI1640 complete media. 1 × 10^5^ monocytes, 2 × 10^5^ monocyte-derived macrophages, or 1 × 10^5^ moDCs were infected with GAS at a multiplicity of infection (MOI) 10 for 1 h. Next, extracellular bacteria were killed by addition of RPMI1640 containing antibiotics (100 µg × mL^−1^ streptomycin, 100 IU × mL^−1^ penicillin G (Hyclone)). After a total of 23 h infection, supernatants were collected and stored at − 80 °C until further analyses. The moDCs were directly prepared for flow cytometry.

For assessing intracellular bacterial survival kinetics, 2 × 10^5^ moDCs were infected, as described above. After addition of antibiotics, the cells were washed, lysed, and intracellular bacteria plated on blood agar plates (Oxoid).

For inhibition studies, moDCs were pre-treated with inhibitors targeting different caspases: caspase-3 (Cas3/7-Inhibitor I (CAS 220509-74-0), Sigma Aldrich, 500 nM; Ac-DEVD-cho (Invivogen), 40 µM), caspase-8 (z-IETD-fmk (Invivogen), 40 µM), or pan-caspase inhibitor (z-VAD-fmk, (Invivogen), 40 µM). moDCs were pre-treated with the respective inhibitors for 1 h at 37 °C and subsequently infected as described above. Inhibition of caspases remained through the entire course of infections. DMSO served as solvent control.

### Isolation of human neutrophils and chemotaxis assay

Human primary neutrophils were isolated from healthy donors using a density gradient centrifugation on Polymorphprep (Axis Shield). Neutrophil viability was assessed via trypan blue staining. After isolation, neutrophils were suspended in RPMI1640 medium (Cytivia) supplemented with 10 mM l-glutamine, 25 mmol/l HEPES (all HyClone), and 5% (v/v) FCS.

Supernatants of uninfected/GAS-infected moDCs or recombinant CXCL8 were added to the basolateral side of a 3 μm pore-size cell culture insert (Corning). 5 × 10^5^ isolated neutrophils were added to the membranes apical side and cells were left to migrate for 2 h at 37 °C. Migrated cells were collected and total numbers were assessed using a Neubauer cell counting chamber.

### Flow cytometry

Dead cells were labeled using the Zombie Aqua Fixable Viability Kit (BioLegend). Unspecific binding of immunoglobulins was blocked by using Human TruStain FcX (BioLegend) according to the manufacturer’s instructions. Incubations of cells with titrated amounts of monoclonal antibodies were carried out for 30 min at 4 °C in the dark. Cells were washed between each staining step and fixed using the Cyto-Fast Fix/Perm Buffer Set (BioLegend). Antibodies and clones directed against the following markers were used (target, clone, fluorochrome, all Bio-Legend): CD209 (9E9A8, APC), CD209 (9E9A8, BV421), CD40 (5C3, Alexa Fluor700), CD80 (2D10, BV711), CD86 (BU63, PE/Cyanine7), CD83 (HB15e, BV421) and HLA-DR (L243, FITC). The gating strategy to identify moDCs is shown in Additional file 1: Figure S1. Macrophages were identified via CD68 and HLA-DR and monocytes via CD14 and CD16 staining as previously described [65]. Data were acquired using a FACSAria III flow cytometer and FACS DIVA 8.0 Software (both BD Biosciences, San Jose, CA, USA) and analyzed using FCS Express 7 Software (De Novo Software).

### Cytokine measurements

Cytokine concentrations in cell culture supernatants were measured via LEGENDPlex human inflammation panel 1 (13-plex) kit or custom panel (3-plex: IL-1β, IL-8, IL18) (both BioLegend) according to the manufacturer’s instructions. Data were acquired with a FACSAria III flow cytometer using FACS DIVA Software (both BD Bioscience) and analyzed using LEGENDPlex software (BioLegend). Furthermore, previously published cytokine levels in acute plasma from NSTI patients from the infect cohort [48] was reanalyzed.

### Caspase activity measurements

Caspase 8 activity was measured using the Caspase-Glo^®^ 8 Assay Systems (Promega) according to manufacturer’s instructions. Caspase 3 activity was measured using the Magic Red Caspase 3/7 Kit (BioRad) according to manufacturer’s instructions.

### Quantitative reverse transcription PCR analysis

Total RNA was isolated using the RiboPure RNA purification Kit (Ambion) according to the manufacturer’s instructions. cDNA synthesis was performed using the Superscript first-strand synthesis system for RT-PCR (Invitrogen). The real-time PCR amplification was performed with an iTaq Universal SYBR Green Supermix kit (Biorad) using a StepOnePlus sequence detection system (Applied Biosystems). The levels of β-actin transcription were used for normalization. Primer sequences are listed in Additional file 1: Table S1.

### Bioinformatic analysis

Analyses of CovR and CovS sequences were performed as described previously [54]. In brief, genes were identified via BLAST (v. 2.9.0 +) using a custom Python script. Mutations were automatically identified using Python (Reference sequence CovS: WP_002991036.1, CovR: WP_002991052.1). Predictions of CovR/S functionality (Additional file 2: Table S2) were based on literature review (Additional file 1: Table S3) or predicted protein structures using AlphaFold2 [30, 31]. Previously published patient cytokine data [48] and whole genome sequences [63] of the GAS strains were retrieved from the INFECT cohort and reanalyzed.

### Proteomic analysis

Infected moDC pellets were resuspended in 100 µl Tris–HCl (5 mM pH 7.4 containing 5% SDS) and immediately disrupted using a Dismembrator (Retsch) at 2,600 rpm for 3 min (in a 4.8 ml Teflon vessel precooled in liquid nitrogen with an 8 mm diameter steel ball). Next, the cell powder was resuspended with 400 µl of preheated (95 °C) Tris–HCl buffer (5 mM, pH 7.4) and the viscous lysate was transferred into a fresh 1.5 ml low bind pre-lubricated Eppendorf tube and shaken for 1 min at 95 °C and 1,400 rpm. The lysate was cooled to RT and 2 µl of a 1 M MgCl_2_ stock solution (final conc. 4 mM MgCl_2_) was added. Next, 1 µl of a 1:100 diluted benzonase (Pierce Universal Nuclease; Pierce) stock solution (final 0.005 U/µl) was added and mixed by short vortexing. The samples were incubated in an ultrasonic bath at RT for 5 min until the viscous lysate was liquefied by complete degradation of DNA and RNA. The raw lysates were centrifuged (17,000 g; RT; 30 min). After centrifugation, the protein lysate was transferred into a fresh 1.5 ml low bind pre-lubricated Eppendorf tube, and the pelleted cell debris was discarded. The protein concentration of the samples was determined using the Micro BCA Protein Assay Kit following the manufacturer’s protocol (Pierce). Samples were stored at − 80 °C until further use. Sample preparation for mass spectrometry measurements was performed using the SP3 protocol as previously described [9].

LC–MS/MS analyses of tryptic peptide solutions were carried out on a reverse phase HPLC chromatography system (Ultimate 3000 nano-LC system, Thermo Fisher Scientific) coupled to a Q Exactive™ Exploris 480 mass spectrometer (Thermo Fisher Scientific) in data-independent acquisition mode. Data analyses were performed in Spectronaut version 17 (Biognosys AG), based on a database search against a Uniprot/Swissprot database limited to 20,375 human entries (version 2022_01) using trypsin/P digest rule with maximum number of two missed cleavages, methionine oxidation as variable modifications (for details see Additional file 1: Table S4). Further DIA-MS data analysis was carried out in R (R Version 4.2.0) using the tidyverse (version 1.3.1), factomineR (version 2.4.0) and PECA package (version 1.32.0) with a paired reproducibility-optimized test statistic (ROTS) [52, 62] as described by Cuypers et al. [17].

### Statistics

Statistical significance of differences was determined using Kruskal–Wallis test with Dunn’s multiple comparison posttest or Mann–Whitney *U* test. Statistics were performed using GraphPad Prism version 8. A *p* value less than 0.05 was considered significant.

## Results

### Reduced release of IL-8 and IL-18 by myeloid cells in response to infections with GAS harboring dysfunctional CovR/S

**Fig. 1:**
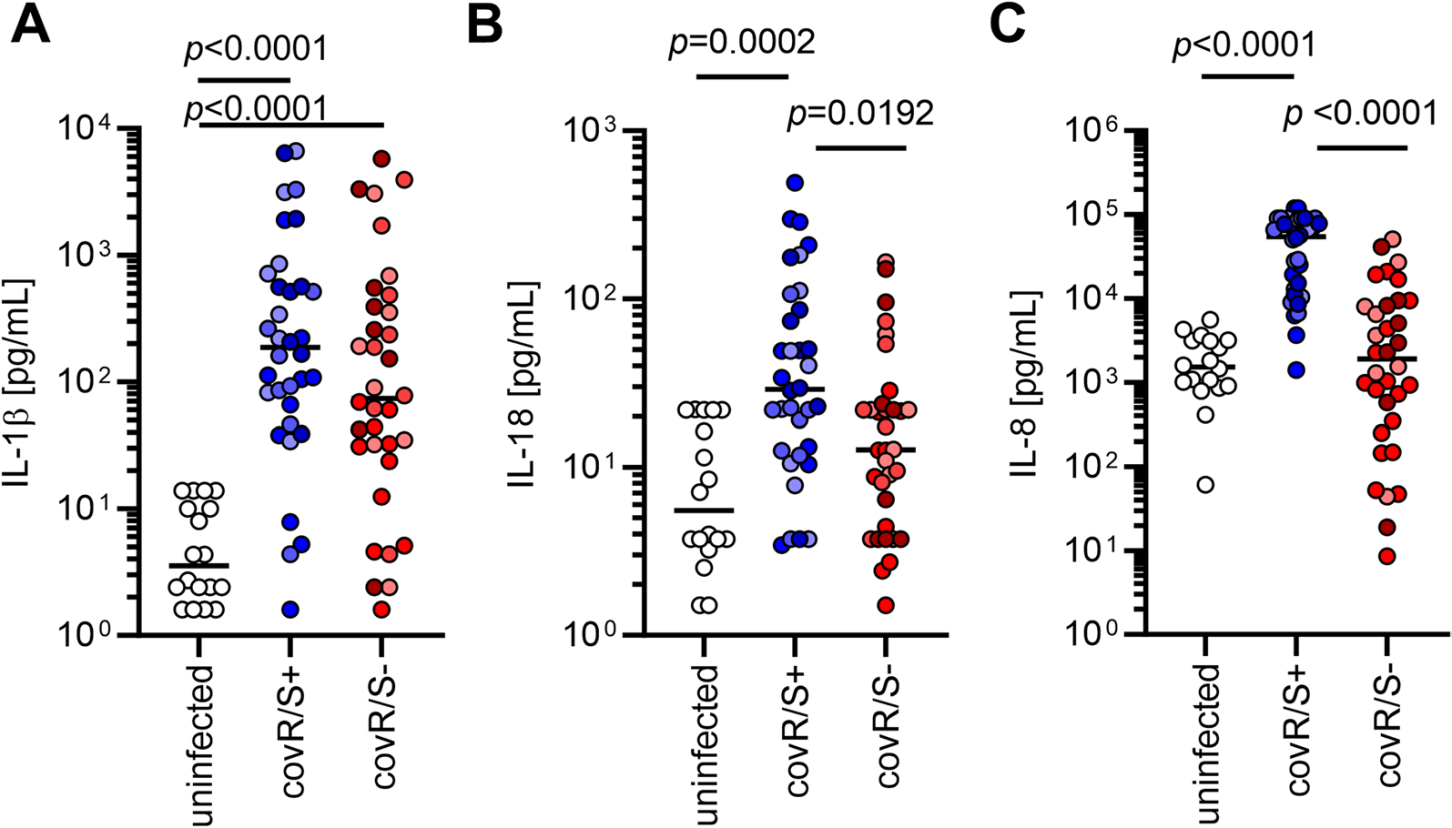
MoDCs infected with GAS strains harboring dysfunctional CovR/S secrete less IL-8 and IL-18. MoDCs were infected with four strains possessing functional CovR/S (5448, 5626, 2006, 8157) or four strains possessing non-functional CovR/S (5448AP, 2002, 5005, 8003) as assessed by sequence analyses and SpeB proteolytic assay. The concentrations of IL-1β (**A**), IL-18 (**B**), and IL-8 (**C**) were measured in supernatants of (un)infected moDCs. Each dot represents one independent experiment with cells from one donor (n ≥ 8). Horizontal lines denote median values. The level of significance was determined using Kruskal–Wallis test with Dunn’s post test. Different shades of color represent infections with different strains

To assess the pro-inflammatory response by moDCs, cells were infected with four GAS strains harboring functional CovR/S (CovR/S^+^; 2006, 5448, 5626, 8157) or four *covS* mutant strains carrying non-functional CovR/S (CovR/S^−^; 2002, MGAS5005, 5448AP, 8003) and IL1β, IL-8 as well as IL-18 release in response to infections was measured. The sequences as well as CovR/S functionality of the respective strains were previously assessed [54] (Additional file 2: Table S2) and confirmed via SpeB proteolytic activity on casein agar plates (Additional file 1: Fig. S2). While moDCs readily secreted equal amounts of IL-1β in response to all infections (Fig. 1A), IL-18 as well as IL-8 levels were significantly less in infections with CovR/S^−^ as compared to CovR/S^+^ strains (Fig. 1B, C).

**Fig. 2:**
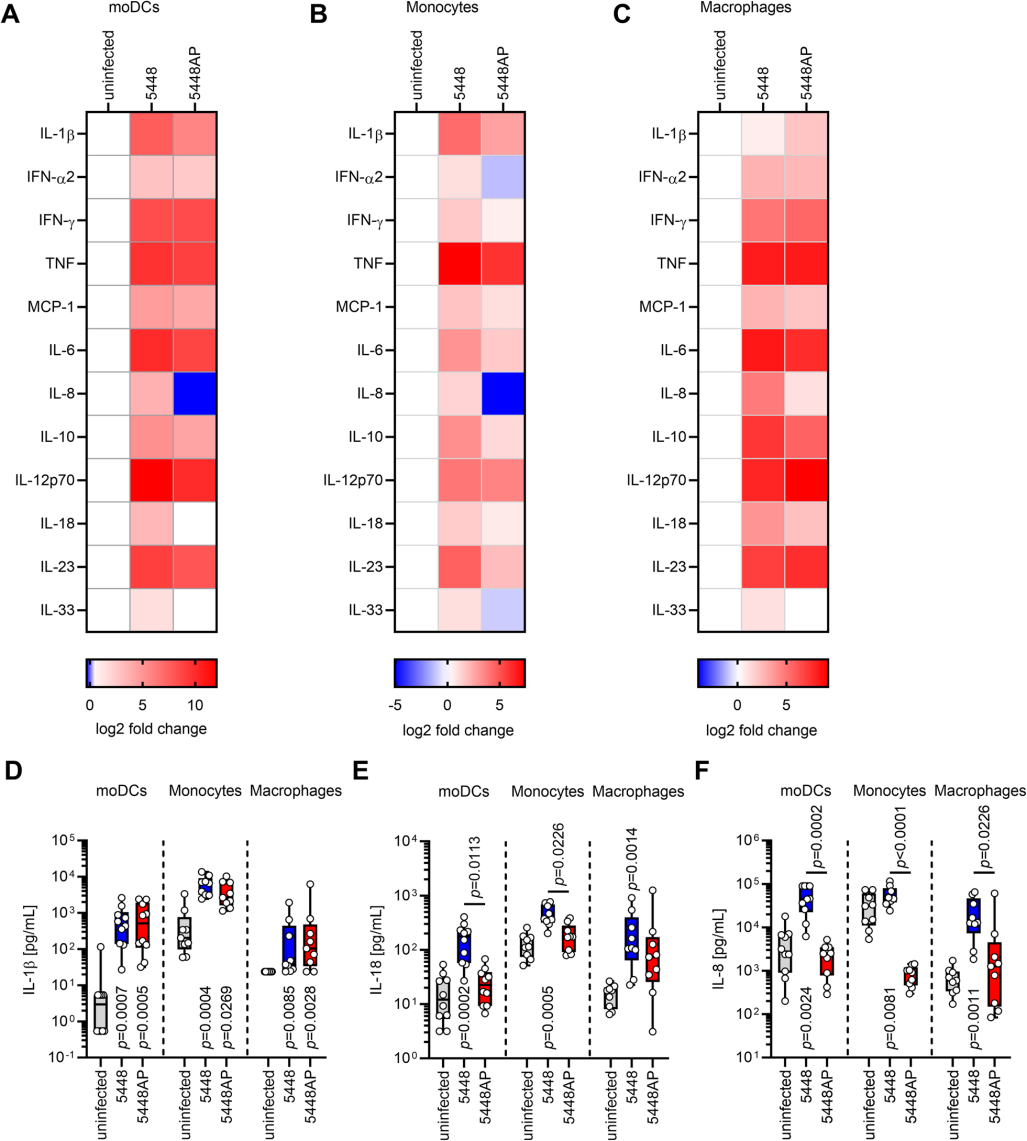
Monocytic cells infected with 5448AP secrete less IL-18 and IL-8. MoDCs (**A**), monocytes (**B**), or monocyte-derived macrophages (**C**) were infected with 5448 or 5448AP and cytokine secretion was measured via a multiplex assay (n ≥ 8). The heatmaps represent the log2 fold change of cytokine concentration in relation to the uninfected controls (**A–C**). Original data are displayed in **D–F** and Additional file 1: Fig. S3. Original data of IL-1β (**D**), IL-18 (**E**), and IL-8 (**F**) concentration in supernatants of (un)infected moDCs, monocytes, and monocyte-derived macrophages. The data in (**D**–**F**) are displayed as box plots. Each dot represents one independent experiment with cells from one donor (n ≥ 8). The level of significance was determined using Kruskal–Wallis test with Dunn’s posttest

To evaluate whether (i) this observation is restricted to moDCs and (ii) to exclude potential impact of genomic variations between the GAS strains, moDCs, monocytes, and monocyte-derived macrophages were infected with *emm*1 STSS isolate 5448 and its corresponding *covS* mutant 5448AP [69] and the release of multiple cytokines in response to infections was assessed (Fig. 2, Additional file 1: Fig. S3). Irrespective of the CovR/S variant used for infections, all three cell types released comparable levels of IFN-γ, tumor necrosis factor (TNF)-α, MCP-1, IL-6, IL-10, IL-12p70, and IL-23 (Fig. 2A–C; Additional file 1: Fig. S3). Notably, of the two inflammasome-related cytokines, only IL-1β release was equally induced (Fig. 2D). In contrast, IL-18 secretion in 5448AP infections remained at the levels of uninfected controls (Fig. 2E). Furthermore, similar results were noted for IL-8 release (Fig. 2F).

### Infected dendritic cells clear intracellular bacteria and mature irrespective of GAS CovR/S phenotype

**Fig. 3:**
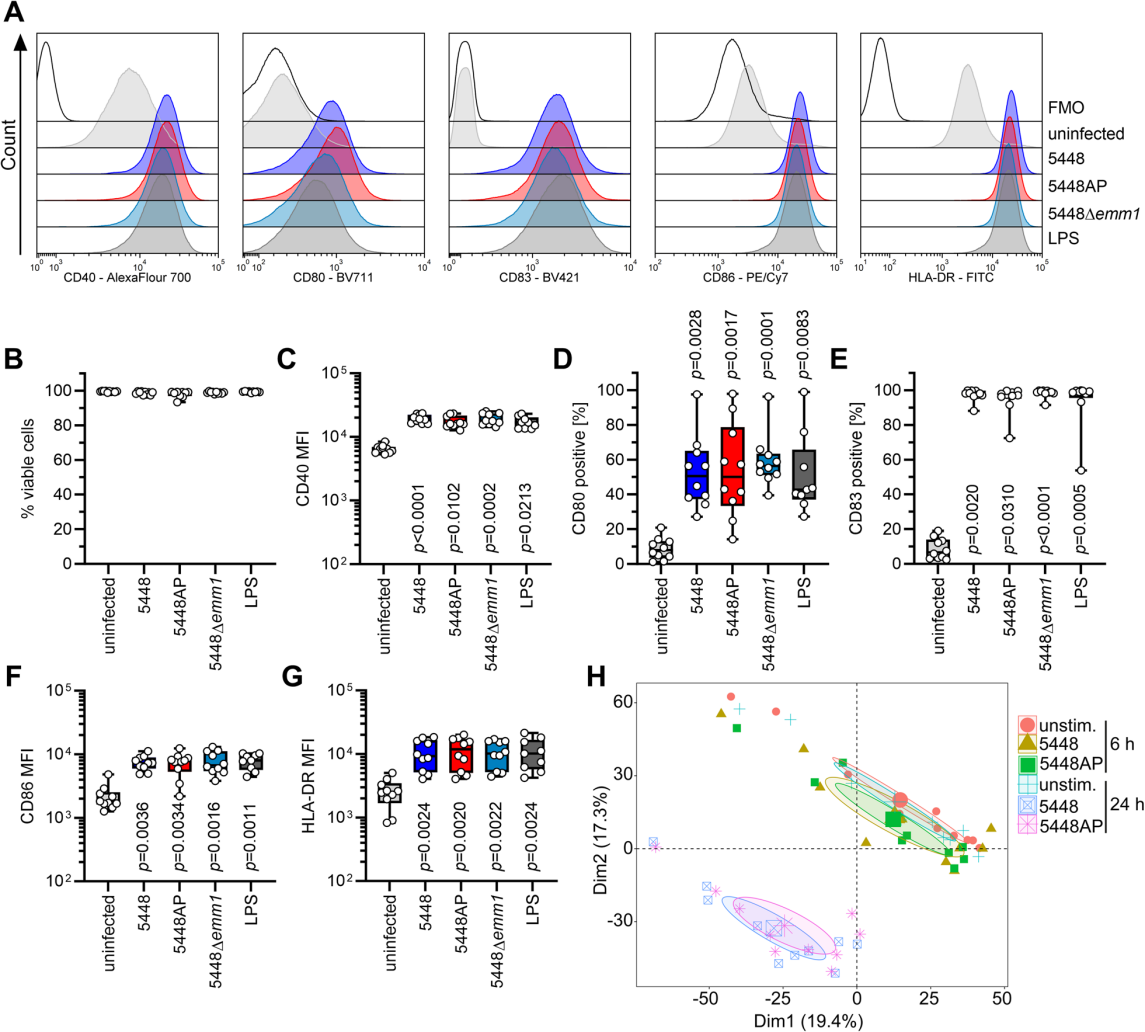
5448AP does not impair DC maturation. MoDC were infected with 5448, 5448AP, or 5448Δ*emm1* for 1 h. Extracellular bacteria were killed by substituting the media with antibiotics for additional 23 h. MoDC phenotype (**A, C–G**) and viability (**B**) were evaluated via flow cytometry. Representative histograms for each marker are shown in (**A**). The maturation process was evaluated by assessing the expression of CD40 (**B**), frequencies of CD80^+^ (**D**) and CD83^+^ (**E**) cells as well as CD86 (**F**) and HLA-DR expression (**G**). **H** Principal component analysis of intracellular proteins 6 h and 24 h post infections with indicated strains. Each dot represents one donor (n = 10). The ellipses indicate the calculated 95% probability region for a bivariate normal distribution with an average center of groups. The data in (**B**–**G**) are displayed as box plots. Each dot represents one independent experiment with cells from one donor (n = 10). The level of significance was determined using the Kruskal–Wallis test with Dunn’s posttest. FMO, fluorescence minus one; MFI, mean fluorescence intensity

Next, we assessed whether 5448AP infections would also have an effect on DC maturation. Since CovR/S inactivation has been reported to increase surface abundance of M-protein [61], an *emm*-knockout strain was included in this series of experiments. MoDCs were infected with 5448, 5448AP, or 5448Δ*emm1* and bacterial intracellular survival (Additional file 1: Fig. S4) and moDC maturation were assessed (Fig. 3A). Irrespective of the strain used, moDCs remained viable (Fig. 3B) and eliminated all bacteria within 24 h of total infection time. No differences in GAS uptake were noted (Additional file 1: Fig. S4). Furthermore, moDCs readily matured in all infectious conditions to the levels of LPS stimulations, characterized by increased frequencies of CD80 and CD83 positive cells as well as upregulation of CD40, CD86, and HLA-DR on the surface (Fig. 3A–G).

To assess potential differences on a global level, whole proteome of moDCs infected with 5448 or 5448AP was quantitatively profiled by mass spectrometry at 6 and 24 h post infection. Uninfected moDCs at respective time points served as controls. In total, 5857 protein groups were identified. For quantification, 4801 protein groups identified by the presence of two or more peptides and observed in at least half of the experiments for each condition were used. Principal component analysis (PCA) revealed that uninfected controls (6 and 24 h) and both 6 h infections with 5448 and 5448AP clustered together (Fig. 3H). Furthermore, rather a donor specific proteome signature with no obvious impact of infections was noted at 6 h (Additional file 1: Fig. S5; Additional file 2: Table S5) suggesting that 6 h of infection are too short to induce maturation. In contrast, the moDC proteomes of both 24 h infections were different from uninfected moDC and separated in the PCA (Fig. 3H). Statistical analyses revealed a general infection specific signature. However, no major differences were observed between 5448 and 5448AP infections (Additional file 1: Fig. S6, Additional file 2: Table S5). Furthermore, IL-18 as well as IL-8 were not detected within moDCs (Additional file 2: Table S5).

### Caspase-8 inhibition restores secretion of IL-8 and IL-18 by moDCs in 5448AP infections

Reduction of IL-8 secretion by action of either SpyCEP [67, 73] or Nga [47] is well established in GAS infections. To exclude potential impact of infections on the transcriptional level, expression of genes *CXCL8*, *IL6,* and IL-8 receptors *CXCR1* and *CXCR2* in moDCs was assessed. While transcription of *CXCR1* remained at the level of uninfected control, *CXCL8*, *IL6* and *CXCR2* transcription was equally increased, irrespective of the strain used (Additional file 1: Fig. S7). In addition, chemoattractant capacity of supernatants derived from infected moDCs towards neutrophils was assessed. Supernatants from both, 5448- and 5448AP-infected moDCs elicited increased migration of neutrophils as compared to the medium control (Additional file 1: Fig. S8).

**Fig. 4:**
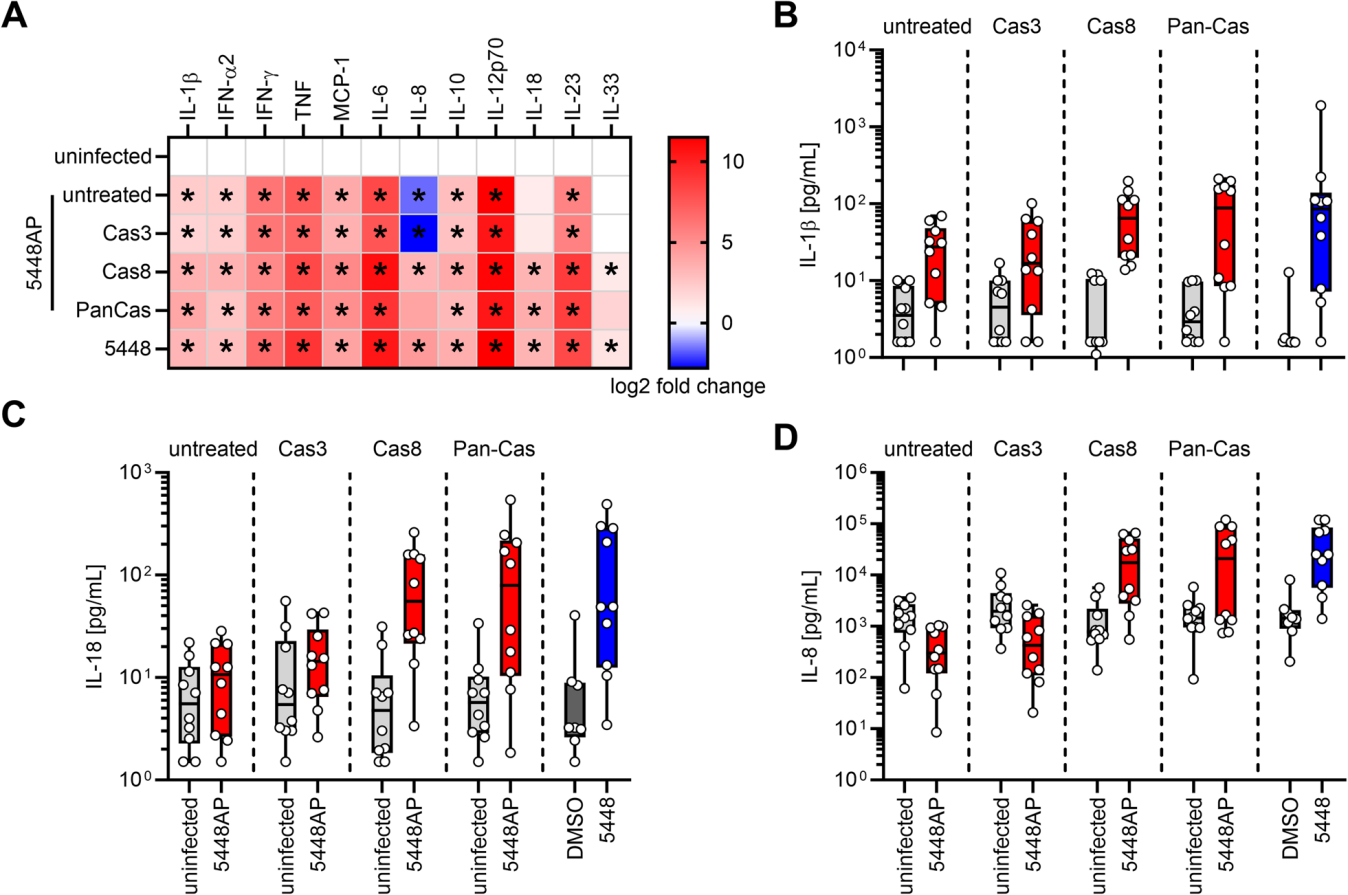
Inhibition of caspase-8 restores IL-18 and IL-8 secretion in 5448AP infections. MoDCs were treated with caspase inhibitors (caspase-3: Cas3/7-Inhibitor I, Ac-DEVD-cho; caspase-8: z-IETD-fmk, pan-capase: z-VAD-fmk) and subsequently infected with 5448AP. Cytokine secretion by moDCs was measured via a multiplex assay. **A** The heatmap represents the log2 fold change of cytokine concentration in relation to the respective uninfected controls. Original data are displayed in **B–D** and Fig. S9. Original data of IL-1β (**B**), IL-18 (**C**), and IL-8 (**D**) concentration in supernatants of (un)infected moDCs. The data in (**B**–**D**) are displayed as box plots. Each dot represents one independent experiment with cells from one donor (n = 10). A separate uninfected control with the respective inhibitor treatment was performed for each 5448AP infection. Untreated: indicates 5448AP infection without inhibitors. Each infection was compared to its respective uninfected controls. The level of significance was determined using Mann–Whitney *U*-test. Exact statistical analysis is displayed in Additional file 1: Table S6

As we did not detect a significant impact of reduced IL-8 levels on neutrophil migration, we further focused on IL-18 secretion by moDCs. The IL-18 amino acid sequence contains predicted cleavage sites for executioner proteases, including caspase-1 and caspase-3 [51]. Processing of IL-18 by caspase-3 was shown to yield biologically inactive fragments [50]. To investigate whether inhibition of caspase-3 or its upstream initiator caspase-8 would restore the moDCs ability to secrete IL-18 (and potentially IL-8), moDCs were pre-treated with specific inhibitors of caspase-3, caspase-8, or a pan-caspase inhibitor. Subsequently, pre-treated moDCs were infected with 5448AP, and multiple inflammatory cytokines were measured in supernatants. No cytokine secretion in response to inhibitors or its solvent, DMSO, was noted (Additional file 1: Fig. S9). Caspase-3, caspase-8, or pan-caspase inhibition did not alter secretion of IFN-α2, IFN-γ, MCP-1, IL-6, IL-10, IL-12p70 or IL-23, as elevated levels of these cytokines were detected in all infection conditions (Fig. 4, Additional file 1: Fig. S9). Furthermore, IL-1β secretion in response to all 5448AP infections remained at the levels of 5448 infections (Fig. 4A, B). Notably, in the presence of caspase-8 as well as pan-caspase inhibitors, the levels of IL-18 increased in response to 5448AP infections, reaching the levels of 5448 infection control (Fig. 4A, C). A similar trend was also observed for IL-8 (Fig. 4A, D), particularly when caspase-8 inhibitor was used.

### Infections of moDCs with 5448AP result in higher caspase-8 activity

**Fig. 5:**
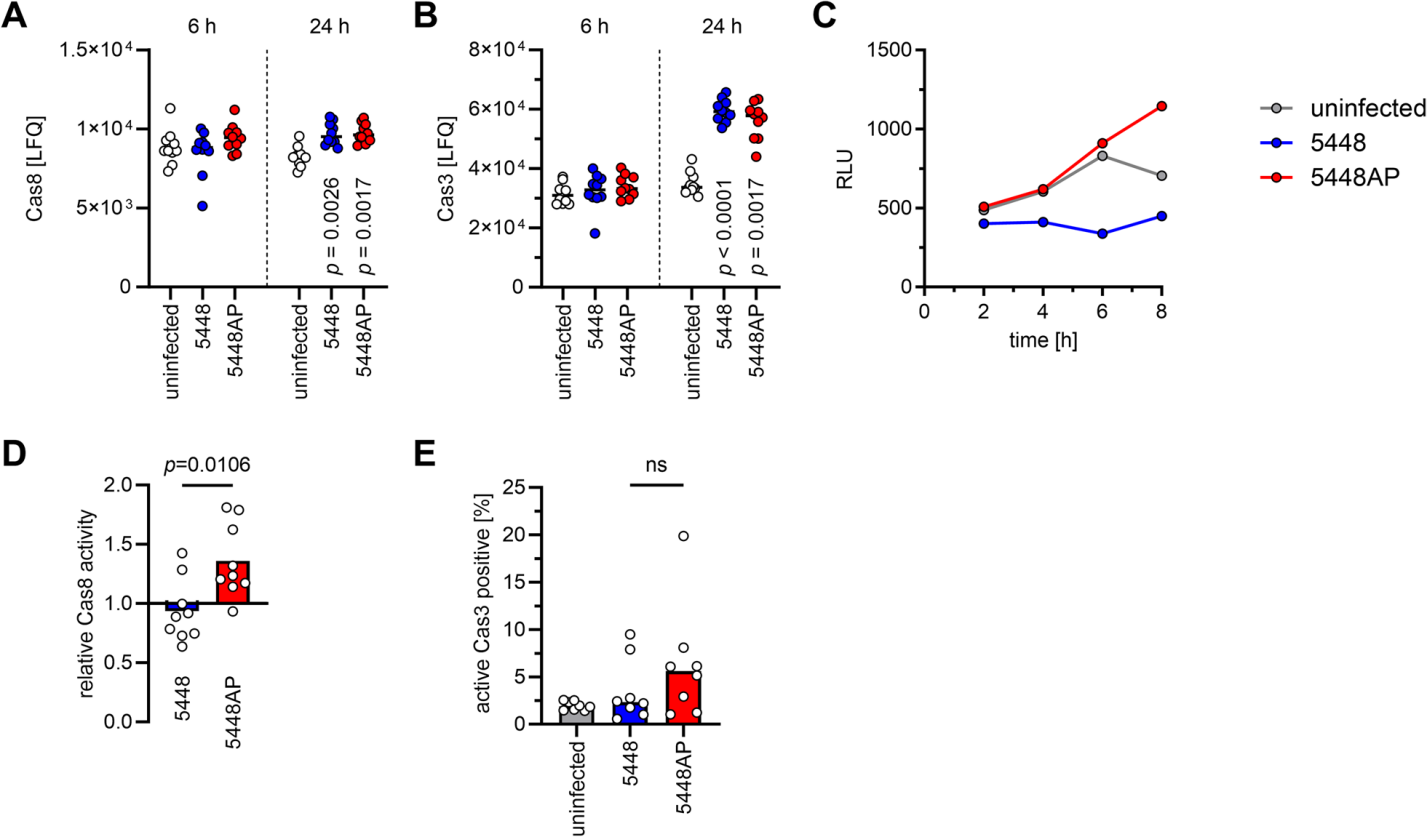
5448AP-infected moDCs exhibit high caspase-8 activity. MoDCs were infected with 5448 or 5448AP. Relative abundance of caspase-8 (**A**) and caspase-3 (**B**) 6 h and 24 h post infection. Original calculations are displayed in Additional file 1: Table S4. **C** Caspase-8 activity of (un)infected moDCs as measured at indicated time points by luminescence. **D** Relative caspase-8 activity at 8 h post infection. **E** Frequencies of caspase-3^+^ moDCs 8 h post infection. Each dot represents one independent experiment with cells from one donor (n ≥ 8). Horizontal lines in (**A, B**) denote median values. Bars in (**D**, **E**) denote mean values. The level of significance was determined using Kruskal–Wallis test with Dunn’s posttest in (**A, B, E**) or Mann–Whitney *U*-test in (**D**). LFQ, label-free quantification intensities; RLU, relative light units

Since caspase-8 inhibition restored the secretion of IL-8 and IL-18 in response to 5448AP infections, its intracellular abundance was assessed in moDCs. Executioner caspase-3 was also analyzed. Irrespective of the strain used for the infections, moDCs equally responded by upregulating the expression of both caspases at 24 h post infection (Fig. 5A, B; Additional file 2: Table S5). However, total protein abundance does not reflect its activity. Therefore, moDCs were infected with both strains and caspase-8 activity was assessed at different time points of infection. Luminescence-based measurement over a period of 8 h revealed a stable increase of caspase-8 activity in response to 5448AP infections (Fig. 5C) In contrast, 5448 infections rather suppressed caspase-8 activity. Assessment of its activity in several donors confirmed the initial result. While 5448AP infections activated caspase-8, 5448 infections did not (Fig. 5D). Downstream assessment of executioner caspase-3 activity via flow cytometry showed that only a negligible percentage of infected moDCs were positive for active caspase-3 (Fig. 5E).

### Impact of dysfunctional CovR/S on systemic cytokine levels in NSTI patients

**Fig. 6:**
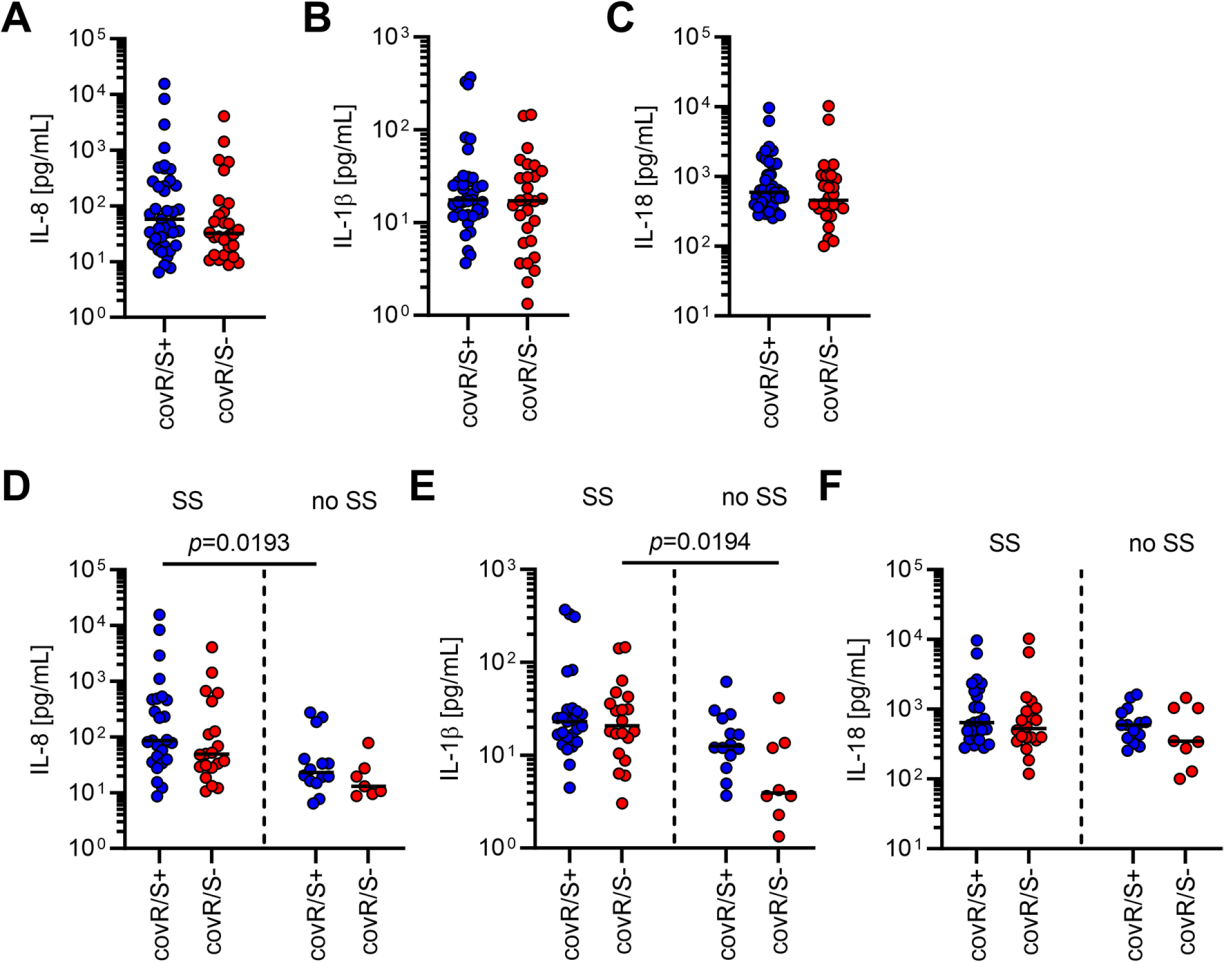
Cytokine/chemokine levels in plasma of NSTI patients. Levels of IL-8 (**A**), IL-1β (**B**), and IL-18 (**C**) were previously determined in plasma of NSTI patients [48] and reanalyzed. Comparison of IL-8 (**D**), IL-1β (**E**), and IL-18 (**F**) plasma concentrations between septic shock and no septic shock. Each dot represents data from one patient. Horizontal lines denote median values. The level of significance was determined using Kruskal–Wallis test with Dunn’s posttest. SS, septic shock

As both, the IL-1β network [14] and the IL-12/IL-18 axis [20] are closely linked to NSTIs and STSS, the CovR/S phenotype of GAS isolates collected during the INFECT study was determined and correlated to previously measured plasma concentrations of IL-8, IL-1β, and IL-18 [48]. In total, *covS* and *covR* genes of 67 isolates were analyzed and amino acid sequences were predicted (Additional file 2: Table S2). The majority of mutations leading to amino acid substitution or truncated proteins were identified in CovS (Additional file 2: Table S2). To assess the functionality of different CovR/S variants, literature review (Additional file 1: Table S3) and protein structure predictions using AlphaFold2 (Additional file 1: Fig. S10) were performed [30, 31]. Twenty-eight strains were predicted to harbor dysfunctional CovR/S (Additional file 2: Table S2, Additional file 1: Fig. S10; please refer to the figure legend of Additional file 1: Fig. S10 for detailed description of the functionality assignment). Next, the CovR/S phenotype was matched to plasma cytokine concentrations of respective patients. Irrespective of functional or dysfunctional CovR/S of the infective GAS, equal systemic concentrations of IL-8, IL-1β, and IL-18 (Fig. 6A–C) were detected in patient’s plasma. As the IL-12/IL-18 axis is also linked to development of STSS, patients were grouped based on clinical presentation, with and without septic shock. These analyses revealed that patients suffering from septic shock have significantly higher systemic IL-8 or IL-1β plasma levels if they are infected with CovR/S^+^ or CovR/S^−^ strains, respectively (Fig. 6D,E). No differences in systemic IL-18 levels between patient groups were observed (Fig. 6F).

### Secretion of IL-1β and IL-18 by moDCs is increased in infections with 5448Δ*slo*

**Fig. 7:**
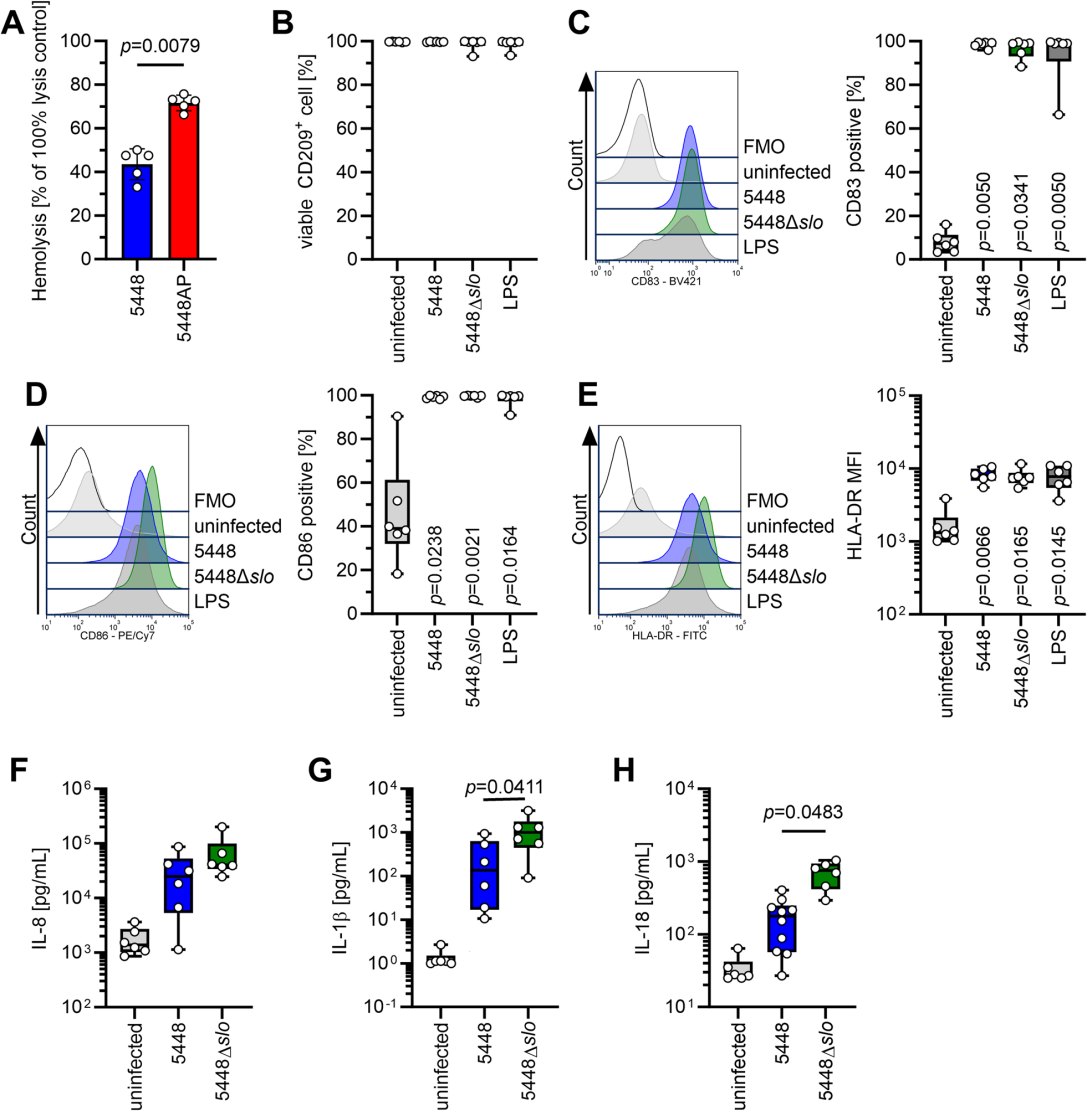
Knock-out of SLO in 5448 leads to elevated secretion of IL-1β and IL-18 by moDCs. **A** SLO hemolytic activity in supernatants of 5448 and 5448AP strains. MoDCs were infected with 5448 or 5448Δ*slo* and moDC viability (**B**) and phenotype (**C–E**) were evaluated via flow cytometry. The maturation process was evaluated by assessing the frequencies of CD83^+^ (**C**) and CD86^+^ (**D**) cells as well as expression of HLA-DR (**E**). Representative histograms for each marker are shown in each respective left panel. The concentrations of IL-8 (**F**), IL-1β (**G**), and IL-18 (**H**) were measured in supernatants of (un)infected moDCs. The data in (**B**–**H**) are displayed as box plots. Each dot represents one independent experiment with cells from one donor (*n* ≥ 6). The level of significance was determined using Kruskal–Wallis test with Dunn’s posttest. FMO, fluorescence minus one; MFI, mean fluorescence intensity

SLO is a known inducer of apoptotic program in myeloid cells, which requires activation of caspase-8 [64]. Since SLO was previously shown to (i) impact maturation of murine DCs, (ii) to interfere with caspases [16, 36], and (iii) its expression is under CovR/S control [60], we hypothesized that SLO might be responsible for impaired secretion of IL-18 by moDCs. First, hemolytic activity of stationary culture supernatants of 5448 and 5448AP was measured. This revealed significantly higher hemolytic activity of 5448AP supernatants as compared to 5448 (Fig. 7A). Several attempts to construct a *slo*-knock-out in 5448AP background was unsuccessful. We therefore hypothesized that infections of moDCs with 5448Δ*slo* strain would further increase IL-18 response as compared to 5448 wild-type infections. Therefore, cells were infected with 5448 and its corresponding 5448Δ*slo*. Deletion of SLO had no obvious impact on moDC viability (Fig. 7B). Furthermore, moDCs matured in response to all infections characterized by increased frequencies of CD83^+^ (Fig. 7C) and CD86^+^ (Fig. 7D) cells as well as increased expression of surface HLA-DR (Fig. 7E). Analyses of cytokines revealed an equal IL-8 response (Fig. 7F). In contrast, IL-1β (Fig. 7G) and IL-18 (Fig. 7H) secretion were significantly higher in 5448Δ*slo* as compared to wild-type infections.

## Discussion

Myeloid cells are crucial responders to infections, responsible for pathogen clearance, recruitment of other immune cells, and shaping the T cell response through secretion of a plethora of cytokines and chemokines. They are the primary sources of IL-1 family cytokines IL-1β and IL-18 as well as potent producers of the main human neutrophil chemoattractant IL-8 [24]. Bacteria, including GAS, have developed multiple phenotypic and genetic adaptations to evade the host immune system effector mechanisms. In this study, we show that infections with GAS strains harboring dysfunctional CovR/S result in reduced secretion of IL-8 and IL-18 by monocytic cells. This phenomenon is mainly mediated by a caspase-8-dependent mechanism.

GAS have developed several ways to adapt to the host environment. Enhanced mutational rates in the *covR/S* genes often mediate the switch to a hyper-virulent phenotype [69]. CovS senses environmental signals and phosphorylates the bound response regulator CovR. Phosphorylated CovR dissociates from CovS, binds to its target DNA sequences, and either activates or represses transcription of target genes [19, 26]. Not every mutation in *covR* or *covS* will lead to dysfunctional TCS [49]. However, loss of function mutations are often associated with increased virulence [18] due to de-repression of transcription of several virulence factors, which is particularly pronounced in *emm1* strains [6, 25, 33]. Of 67 strains analyzed in this study, 28 isolates possessed mutations, which were experimentally validated or predicted to negatively affect the functionality of the TCS resulting in a hyper-virulent phenotype. In such strains, increased levels of key virulence factors, including the capsule, M protein, SpyCEP, and Nga were reported [60, 61].

The cell-envelope protease SpyCEP was shown to cleave human IL-8, resulting in decreased local levels and consequently impaired neutrophil recruitment to the site of infection [56, 67, 73]. However, reduction of IL-8 levels in CovR/S^−^ infections of moDCs did not affect neutrophil migration, likely due to the presence of other chemoattractants. Induction of ER stress and Golgi fragmentation by the NAD-glycohydrolase Nga was shown to reduce secretion of IL-8 by GAS-infected macrophages [47]. Furthermore, Nga activity inhibits type I interferon production in macrophages via suppression of STING signaling, which also correlates with increased disease severity in NSTI patients [44]. The results of this study are in line with previous observations. However, our data indicates that streptococcal SpyCEP and Nga are not the only factors responsible for the abrogated IL-8 secretion because (i) NF-κB and other pro-inflammatory signaling pathways are equally affected in all infectious conditions and (ii) inhibition of initiator caspase-8 restored IL-8 release by moDCs. This suggests that particularly host factors play a role in the observed phenotype, which is potentially independent of gene regulation.

Strains harboring dysfunctional CovR/S were also shown to produce increased levels of SLO [36, 61]. SLO forms pores in membranes of phagocytes [72], mediates bacterial escape from phagosomes, and induces macrophage death [64]. In addition, SLO is essential for Nga translocation across the plasma membrane into the host cell cytosol. Consequently, Nga depletes cellular energy stores and interferes with cytokine secretion [44, 47]. STSS isolates were shown to produce significantly more SLO than strains derived from non-invasive infections, emphasizing a crucial role for this cytolysin in invasive diseases [8, 27]. Much of the knowledge about DC-GAS interactions is derived from work with murine cells or prolonged infection times. In such settings, DCs infected with a CovR/S dysfunctional and high-SLO secreting 5448AP strain showed impaired maturation and significant cell death [16, 36]. Our results are in stark contrast to these previous findings mostly due to the following reasons: (i) short initial infections which were not exceeding one hour and (ii) immediate depletion of extracellular bacterial supply through antibiotic treatment. Even in such setting, IL-18 secretion by infected monocytic cells was impaired. SLO is a known inducer of apoptotic program through activation of the initiator caspase-8 [64]. Although speculative, increased production of SLO by phagocytosed GAS might lead to the observed increased activation of caspase-8. Consequently, caspase-8 or a downstream-protease other than caspase-3 might cleave IL-18, which results in the observed abrogated IL-18 secretion. Although we were not able to construct a *slo*-knockout in 5448AP background, our data supports the concept that SLO interferes with the cytokine axis, since moDCs infected with 5448Δ*slo* secreted high amounts of IL-1β and IL-18, even exceeding the levels of wild-type infections.

The role of the IL-1β network in NSTIs is well established [14]. Furthermore, the IL-12/IL-18 axis has been linked to the pathological MAIT cell activation and development of STSS [20, 46, 48]. Although we did not detect differences in systemic IL-18 levels in patients, local tissue concentrations might differ due to a mix of GAS clones, which are usually recovered from NSTI tissue biopsies [55]. Reduced local IL-18 levels could potentially delay activation of resident or recruited MAIT cells characterized by reduced IFN-γ production. Nonetheless, hyper-virulent GAS harboring *covR/S* mutations produce increased amounts of superantigens [61], which might potentially negate the IL-18 effect. In general, myeloid cells are the primary sources of IL-1β and IL-18 [24] and elevated levels of both cytokines were found locally and systemically in a pharyngitis human challenge trial as well as increased frequencies of circulating monocytes and DCs [3]. In our *in vitro* experimental setup, strains harboring dysfunctional CovR/S exclusively suppressed IL-18 release by myeloid cells. Notably, these two cytokines are commonly considered to be processed and released together. Thus, little is known about their differential processing. Canonical maturation and release are mediated by inflammasome-associated executioner caspase-1. However, other non-canonical inflammasomes as well as proteases can process both cytokines [71]; e.g., caspase-8 is able to substitute for loss of caspase-1 dependent processing of IL-18 [4, 10, 22, 23, 42]. In addition, pyroptosis or autophagy mediate caspase-1 and/or caspase-8 dependent release of both cytokines and blocking of caspase-8 even further enhances the release [68, 70]. However, the overall moDC cell death was not affected by the CovR/S phenotype. Furthermore, cleavage of pro- and mature IL-18 via caspase-3 was shown to yield biologically inactive fragments [1, 50]. In this study, inhibition of caspase-8 restored secretion of IL-18 in infections with hyper-virulent GAS, while inhibition of caspase-3 did not. CovR/S controls up-to 15% of the GAS genome. Mutations in this TCS might lead to loss of certain virulence factor (e.g., SpeB) and overproduction of others (e.g., SLO) [53]. SpeB can cleave both, pro-IL-1β [37] and IL-18 [29], into the respective mature cytokines and therefore substitute for caspase actions. Furthermore, SpeB is responsible for proteolytic cleavage of autophagy related host proteins [7]. However, these mechanisms do not apply to infections with CovR/S-negative strains, since they do not produce SpeB. Although speculative, our data supports the concept that (i) IL-18 expression and/or processing can occur independent of IL-1β and (ii) either active caspase-8 or a yet unknown host factor downstream is responsible for the observed effect, which is also supported by the fact that caspase-8 activity was exclusively increased in 5448AP infections.

## Conclusions

Cytokines/chemokines derived from myeloid cells are crucial effector molecules in infections. Through tissue passage, GAS adopt a hyper-virulent phenotype mediated by mutations of genes encoding the CovR/S TCS. Here, we show that *in vitro* infections of monocytic cells with such hyper-virulent strains result in abrogated release of IL-8 and IL-18. To our knowledge, this is the first report that provides evidence of IL-18 suppression by hyper-virulent GAS strains in cells of monocytic lineage and warrants further experimental studies to identify host as well as bacterial factors, which are used to interfere with the immune response.

### Supplementary Information

**Additional file 1: Figure S1.** Gating strategy used to identify human moDCs. Doublets were excluded by consecutive gating of FSC‐H/FSC‐W and SSC‐H/SSC‐W. MoDCs were selected based on the expression of the specific moDC marker DC‐SIGN (CD209). Dead cells were excluded by using the Zombie Aqua™ Fixable Viability Kit. **Figure S2.** CovR/S TCS functionality as assessed via SpeB proteolytic activity on casein agar plates. Presence of hydrolysis zones around the colonies was categorized as CovR/S^+^. Lack of hydrolysis zones was categorized as CovR/S^−^. **Figure** **S3.** MoDCs, monocytes, or monocyte-derived macrophages were infected with 5448 or 5448AP and cytokine secretion by myeloid cells was measured via a multiplex assay (n ≥ 8). Displayed are concentrations of IFN-α2 (A), IFN-γ (B), TNF-α (C), MCP-1 (D), IL-6 (E), IL-10 (F), IL-12p70 (G), IL-23 (H), and IL-33 (I) in supernatants of (un)infected cells. The data are displayed as box plots. Each dot represents one independent experiment with cells from one donor (n ≥ 8). The level of significance was determined using Kruskal–Wallis test with Dunn’s posttest. **Figure S4.** MoDCs eliminate intracellular GAS. Viable intracellular bacteria were determined at indicated time points post infections. Each dot represents one independent experiment with cells from one donor (n ≥ 10). Horizontal lines denote median value. **Figure** **S5.** Heatmap (z-scored) depicting protein intensities (maxLFQ) in moDCs 6 h post infection. Each column represents one condition for one respective donor (n = 10). In total, 5857 protein groups were identified. For quantification 4801 protein groups, identified with two or more peptides and found in 50% of the individual condition replicates, were used. **Figure S6.** Heatmap (z-scored) depicting protein intensities (maxLFQ) in moDCs 24 h post infection. Each column represents one condition for one respective donor (n = 10). In total, 5857 protein groups were identified. For quantification 4801 protein groups, identified with two or more peptides and found in 50% of the individual condition replicates, were used. **Figure S7.** Gene expression analyses in infected moDCs. Human primary moDCs were infected with GAS 5448 or 5448AP (MOI 10). Extracellular bacteria were killed by substituting the media with antibiotics. Gene expression of genes encoding for CXCL8, IL-6, CXCR1 and CXCR2 relative to uninfected controls (dashed line) are shown. The data are displayed as box plots. Each dot represents one independent experiment with cells from one donor (n = 5). **Figure S8.** Chemotactic effects of infection supernatants derived from 5448/5448AP infected moDCs on primary neutrophils. Neutrophil migration was determined using a transwell assay system. Neutrophils were left to migrate against supernatants of (un)infected moDCs or 12.5 ng × mL^−1^ IL-8 for 2 h. Migration of neutrophils from four donors (n = 4) was measured against conditioned culture medium derived from (un)infected moDCs from 2 individual donors. RPMI media was used as a medium control to assess spontaneous migration of neutrophils. **Figure S9.** MoDCs were treated with caspase inhibitors (caspase-3: Cas3/7-Inhibitor I, Ac-DEVD-cho; caspase-8: z-IETD-fmk, pan-capase: z-VAD-fmk) and subsequently infected with 5448AP. Cytokine secretion by moDCs was measured via a multiplex assay. The concentrations of IFN-α2 (A), IFN-γ (B), TNF-α (C), MCP-1 (D), IL-6 (E), IL-10 (F), IL-12p70 (G), IL-23 (H), and IL-33 (I) were measured in supernatants of (un)infected moDCs. The data are displayed as box plots. Each dot represents one independent experiment with cells from one donor (n = 10). A separate uninfected control with the respective inhibitor treatment was performed for each 5448AP infection. Untreated: indicates 5448AP infection without inhibitors. Each infection was compared to its respective uninfected controls. The level of significance was determined using Mann–Whitney U-test. Exact *p*-values are displayed in Table S6. **Figure S10. **Structural analyses of the overall fold of the CovS/CovR histidine kinase response regulator complex by AlphaFold2.[32,33]. (A) AlphaFold2 was used to predict the overall structure of the tetrameric CovR_2_•2CovS complex. As described for two-component signaling systems, the histidine kinase CovS dimerizes. It consists of a periplasmic sensor-domain (SD), a section that contains the transmembrane helices, a HAMP (histidine kinases, adenylyl cyclases, methyl-accepting chemotaxis proteins and phosphatases)-linker-domain forming a four-helix bundle, a dimerization and histidine phosphotransfer (DHp)-domain containing the phosphorylated histidine residue (H280) and the histidine-kinase (HK)-domain containing the ATP-binding site. The response regulator CovR binds to the DHp-domain and HK-domain. Two molecules of CovS bind to a CovR-dimer. CovR is phosphorylated at an aspartate residue, D53, that induces dissociation from CovS to bind to the DNA. The structure shows that CovS is in a state incompatible with phosphorylation of H280 and transfer of the phosphoryl-group to D53, suggesting that activation of the sensor-domain by ligand binding, such as LL-37, or by a pH shift into the acidic range. The figure was created by PyMOL.[44]. (B) The sensor domain (SD) of the histidine kinase CovS dimerizes by formation of interactions of residues from chain A with residues of chain B. M125 and R104 were found to be mutated to isoleucine and histidine, respectively. Mutation of R104H would interfere with formation of several interactions. R104 from chain B/A forms an important salt bridge with the side chain of D115 of chain A/B, i.e. *in trans*, thereby interconnecting the dimer. Moreover, R104 of chain B/A forms hydrogen bonds to the main chain carbonyl of A112 and D111 of chain A/B, respectively. Asp115 is part of a large electrostatic network containing several acidic residues (right panel). The side chain of Asp115 shows an increased pK_a_ value of 6.09 for chain A and of 5.85 for chain B, as determined by the APBS-PDB2PQR software suite.[34] This would allow alteration of the protonation state dependent of the pH and thereby to translate changes in the pH-value into altered conformations. It is postulated that CovS senses acidic shifts of the environmental pH to approximately pH 5.5. Upon protonation of the Asp115 side chains of chainA/B, formation of the salt bridge with R104 in chain B/A is abolished. This might result in alteration of the sensor-domain conformation that is further propagated into the cell, finally activating the histidine kinase activity. The impact of the mutation of M125I is less obvious. It might interfere with the conformation of the SD. The pK_a_-values were determined by the APBS-PDB2PQR software suite.[34] The figure was created by PyMOL.[44]. (C) The surface of the sensor-domain contains a strong acidic patch. The electrostatic surface potential is shown from -5kT/e (negatively charged, red) to + 5kT/e (positively charged, blue). D115 of chain A is part of this acidic patch visible in this representation. The acidic patch of chain B is on the opposite surface area. The figure was created by the APBS plugin in PyMOL.[44]. (D) Residues that are mutated in CovS cluster at the transition from the HAMP-domain to the DHp-domain. The residues E226, M228, and Q4 were found to be mutated, and patch 163-KLET-266 was found to be deleted. This area is important to translate the extracellular activation of the sensor-domain (SD) into the activation of the histidine kinase (HK). To this end, a conformational change might be elicited that results in the rotation of the -helices of the HAMP- and DHp-domains that bring H280 located in DHp-domain in proximity to the ATP-bound to the HK-domain. Mutation of residues in this area might impair the signal-transduction from the sensor-domain to finally activate the histidine kinase. The figure was created by PyMOL.[44]. (E) Structural organization of the histidine kinase domain. The CovS HK-domain shows all sequence motifs essential for an active histidine kinase. The D-box contains an aspartate, i.e. D425, that directly contacts the exocyclic amino group of the adenine base at C6, thereby creating specificity for adenine nucleotides. The F-box as a central element contains F438. The ATP-lid covers the ATP-molecule. The G-box contains several glycine side chains. This sequence element contacts the - and -phosphates and is similar to a P-loop described for other nucleotide binding proteins. It is needed for ATP-binding and forms an oxyanion hole to stabilize a negative charge occurring at the phosphate oxygen during catalysis. G461 within the G-box was found to be mutated to serine. This mutation might directly interfere with ATP/ADP-binding and might impair phosphoryl-group transfer. The stretch from E418 to K432 was found to be deleted. This will render the kinase inactive, as ATP/ADP-binding will be impaired and the HK-fold will be disrupted. Finally, the N-box contains a conserved asparagine, i.e. N396. N396 is involved in coordinating the bound Mg^2+^-ion needed for nucleotide binding and to allow the nucleophilic attack of the activated H280 for phosphoryl-group transfer. Mg^2+^-ATP was modelled by superimposing the CovS•CovR structure and the structure 4KP4 by PyMOL.[44] K399 of the N-box forms a salt bridge with D327 of the DHp-domain, thereby positioning the HK-domain in the conformation analyzed here. D327 within the DHp domain was found to be mutated to asparagine. Related histidine kinases have a glutamine at the analogous position. As asparagine and glutamine have similar physicochemical properties but are sterically different, an impact of CovS•CovR function cannot be excluded. Another mutation identified in the HK-domain is K498N at the C-terminus. This residue is solvent-exposed and might not directly affect CovS activity. K is a positively charged residue at physiological pH and might be important for solubility of CovS. Furthermore, lysines were known to be post-translationally modified, i.e. by lysine acetylation. Mutation of K498N would abolish this. The figure was created by PyMOL.[44]. (F) Closeup of the CovS DHp-domain showing position of I332 and the phosphoryl-group acceptor H280 and R283. I332 was found to be mutated to valine. Although this is an exchange of residues with similar physicochemical properties, both being hydrophobic side chains, they are sterically different. As predicted by marcoil I332 is positioned at position “a” of the coiled-coil heptad repeat [20]. This means that it is directly involved in the formation of the interface of the coiled-coil structure. Exchange of I332 to V, therefore, might affect the coiled-coil structure. As a direct consequence this could affect the position of the phosphoryl-group acceptor H280 and E281 serving as hydrogen bond acceptor orienting and activating H280 for nucleophilic attack on the -phosphate of the ATP molecule. Notably, H280 shows a strongly reduced pK_a_ value of 2.09 (chain A)/2.10 (Chain B) compared to the free amino acid. This shows that H280 is deprotonated in this structural environment and able to act as a strong nucleophile during catalysis. R283 is postulated to be important for phosphoryl-group transfer by contacting the negatively-charged ATP-phosphoryl groups. Similar to an arginine-finger in small GTP-binding proteins, this might improve the nucleophilic attack of H280 on the -phosphate by neutralizing the negative charge emerging in the transition state of catalysis. Besides, R383 contacts H280. Deletion of R283 affects the pK_a_ of H280, shifting it to 5.24 (chain A)/5.18 (chain B). This shows that deletion of R283 decreases nucleophilicity of H280. R283 was found to be deleted in CovS variants. While AlphaFold2 structure predictions (not shown) suggest that deletion of R283 does not affect CovS structure, it will impair catalytic activity of HK. The pK_a_-values were determined by the APBS-PDB2PQR software suite.[34] The figure was created by PyMOL.[44]. (G) Structure of the response-regulator CovR bound to histidine kinase CovS. Residues found to be mutated in CovR are shown in dark red. R36 and V128 are positioned towards the solvent. It was found that R36 is mutated to cysteine. Mutation of a solvent exposed residue to cysteine might affect protein function by formation of cysteine disulfide bonds, if occurring under non-reducing conditions. M170 is solvent exposed but might also play a structural role. i.e. as hydrophobic amino acid and by acting hydrogen bond acceptor via bridging water molecules not visible in this structural model. T80 was found to be mutated to alanine. T80 is located not too far away from D53, the phosphoryl-group acceptor site in CovR. The hydroxyl-group at the side chain of T80 might play a structural role, i.e. via formation of hydrogen bonds with bridging water molecule. This cannot be achieved by an alanine side chain. If this manifests in CovS•CovR activity needs to be evaluated. R203 and Y198 are located within the CovR DNA-binding helix. In the CovS-bound state R203 is solvent exposed and Y198 might play a structural role via formation of hydrogen bonds between the side chain hydroxyl and bridging water molecules. The figure was created by PyMOL.[44]. (H) Superposition of the AlphaFold2 CovR•CovS structure with the structure of the response regulator CheY (PDB: 2CHE) suggests that the response-regulator CovR binds Mg^2+^. The Mg^2+^-binding site is totally conserved. The Mg^2+^ is hexacoordinated by three water molecules (w1, w2, w3), the side chain of D110, the main chain carbonyl oxygen of M55 (N59 in CheY) and by the side chain of the phosphoryl-group acceptor D53. E9 positions w2 by formation of a hydrogen bond. K102 contacts D53 by formation of a salt bridge. During catalysis the carboxylate of D53 would act as a nucleophile and performs an in-line attack to the phosphoimidazol ring of H280 with H280 acting as a leaving group. It is suggested that the bound Mg^2+^ stabilizes the pentavalent bipyramidal transition state during phosphoryl-transfer reactions. In analogy to CheY, K102 in CovR might be dispensable for the phosphoryl-transfer reaction. However, it might bind to the phosphorylated D53 carboxylate thereby eliciting conformational changes. This might result in dissociation of CovR from CovS adopting a conformation that allows CovR to bind to the target DNA sequence. The figure was created by PyMOL[44]. (I) Overall conformation of the non-complexed, non-phosphorylated CovR dimer as predicted by AlphaFold2. CovR can be separated in a C-terminal DNA-binding domain (aa129-227) with the DNA-binding helix containing R203 and Y198 and a N-terminal receiver (REC)-domain (4–117) that contains D53 that is phosphorylated by the histidine kinase of CovS in order to act as a transcriptional regulator. The conformation shown here is not compatible with DNA-binding at both DNA-binding sites. The figure was created by PyMOL.[44]. (J) Closeup of the DNA-binding site of CovR in complex with DNA. CovR was superimposed with 1GXP. CovR inserts an -helix into the major groove of the DNA. R203 and Y198 form contacts to the sugar-phosphate backbone. The side chain hydroxyl of Y198 forms a hydrogen bond with the phosphate of the DNA-backbone, R203 forms a salt bridge with the phosphate of the DNA-backbone. In this conformation, both residues would contribute to DNA-binding affinity but not to create sequence specificity. However, binding to DNA might alter the conformation and a crystal structure of CovS in complex with dsDNA would be needed to judge this. R203 was shown to be mutated to serine, which is not compatible with creating the interactions with DNA. Y198 was found to be deleted in CovR variants. Mutation of R203S will likely decrease binding affinity while possibly retaining some DNA-binding. However, deletion of Y198 will most likely result in variant not capable to bind DNA as all residues that mediate DNA-binding, i.e. R200, R203 and K205, are located C-terminally of Y198 and will not be properly oriented to allow DNA-binding. The figure was created by PyMOL [44]. **Table S1.** qRTPCR primer used in this study. **Table S3.** Literature review on previously characterized *covR/S* mutations*.* **Table S4.** Parameters for mass spectrometry. **Table S6.** Exact *p*-values of statistical analysis of cytokine secretion by moDCs after caspase-inhibition (n = 10). A separate uninfected control with the respective inhibitor treatment was performed for each 5448AP infection. The level of significance was determined using Mann–Whitney *U*-test.

**Additional file 2: Table S2.** CovR/S phenotype of GAS isolates recovered from the INFECT cohort. Type of infection (poly-/monomicrobial), involvement of septic shock, CovR/CovS mutations as identified by bioinformatics, and CovR/S functionality. Data was published in [51] and re-analyzed based on CovR/S functionality. CovR/S functionality was assessed by literature review (Table S2) and protein structure prediction (Fig. S10). Yellow color indicates dysfunctional CovR/S system. **Table S5.** Statistical analyses of the abundance patterns of proteins of (un)infected moDCs 6 h and 24 h post indicated infections. Color code: blue: log_2_ fold change below 0; red: log_2_ fold change above 0.

## Data Availability

All data associated with this study are presented in the manuscript and supplementary material. Whole genome sequencing data of GAS strains are available at the European Nucleotide Archive (ENA) under the reference number PRJNA524111. The mass spectrometry proteomics data have been deposited to MassIVE with the dataset identifier MSV000093585 (https://doi.org/10.25345/C5154F06B).

## Abbreviations

GAS: Group A streptococcus
NSTI: Necrotizing skin and soft tissue infection
TCS: Two component system
STSS: Streptococcal toxic shock syndrome
SLO: Streptolysin O
(mo)DC: (Monocyte-derived) dendritic cell
IL: Interleukin
IFN: Interferon
MAIT: Mucosa associated invariant T cell
CD: Cluster of differentiation
