# Supplementary material for: Reduced interleukin-18 secretion by human monocytic cells in response to infections with hyper-virulent Streptococcus pyogenes
Source: J Biomed Sci. 2024 Feb 27;31:26. doi: 10.1186/s12929-024-01014-9 (PMC10898077; doi:10.1186/s12929-024-01014-9)
Supplement: Supplementary file 1 — Additional file 1: Figure S1. Gating strategy used to identify human moDCs. Doublets were excluded by consecutive gating of FSC‐H/FSC‐W and SSC‐H/SSC‐W. MoDCs were selected based on the expression of the specific moDC marker DC‐SIGN (CD209). Dead cells were excluded by using the Zombie Aqua™ Fixable Viability Kit. Figure S2. CovR/S TCS functionality as assessed via SpeB proteolytic activity on casein agar plates. Presence of hydrolysis zones around the colonies was categorized as CovR/S+. Lack of hydrolysis zones was categorized as CovR/S−. Figure S3. MoDCs, monocytes, or monocyte-derived macrophages were infected with 5448 or 5448AP and cytokine secretion by myeloid cells was measured via a multiplex assay (n ≥ 8). Displayed are concentrations of IFN-α2 (A), IFN-γ (B), TNF-α (C), MCP-1 (D), IL-6 (E), IL-10 (F), IL-12p70 (G), IL-23 (H), and IL-33 (I) in supernatants of (un)infected cells. The data are displayed as box plots. Each dot represents one independent experiment with cells from one donor (n ≥ 8). The level of significance was determined using Kruskal–Wallis test with Dunn’s posttest. Figure S4. MoDCs eliminate intracellular GAS. Viable intracellular bacteria were determined at indicated time points post infections. Each dot represents one independent experiment with cells from one donor (n ≥ 10). Horizontal lines denote median value. Figure S5. Heatmap (z-scored) depicting protein intensities (maxLFQ) in moDCs 6 h post infection. Each column represents one condition for one respective donor (n = 10). In total, 5857 protein groups were identified. For quantification 4801 protein groups, identified with two or more peptides and found in 50% of the individual condition replicates, were used. Figure S6. Heatmap (z-scored) depicting protein intensities (maxLFQ) in moDCs 24 h post infection. Each column represents one condition for one respective donor (n = 10). In total, 5857 protein groups were identified. For quantification 4801 protein groups, identified with [file 12929_2024_1014_MOESM1_ESM.pdf]

Additional File 1

Reduced interleukin-18 secretion by human monocytic cells in response to infections with hyper-virulent *Streptococcus pyogenes*

Lea A. Tölken[1], Antje D. Paulikat[1], Lana H. Jachmann[1], Alexander Reder[2], Manuela Gesell Salazar[2], Laura M. Palma Medina[3], Stephan Michalik[2], Uwe Völker[2], Mattias Svensson[3], Anna Norrby-Teglund[3], Katharina J. Hoff[4], Michael Lammers[5], and Nikolai Siemens[1#]

[1]Department of Molecular Genetics and Infection Biology, University of Greifswald, Greifswald, Germany

[2]Department of Functional Genomics, University Medicine Greifswald, Greifswald, Germany.

[3]Center for Infectious Medicine, Karolinska Institutet, Karolinska University Hospital, Huddinge, Sweden

[4]Institute of Mathematics and Computer Science, University of Greifswald, Greifswald, Germany

[5]Department of Synthetic and Structural Biochemistry, Institute of Biochemistry, University of Greifswald, Greifswald, Germany

[#]Correspondence: Nikolai Siemens; Email: nikolai.siemens@uni-greifswald.de

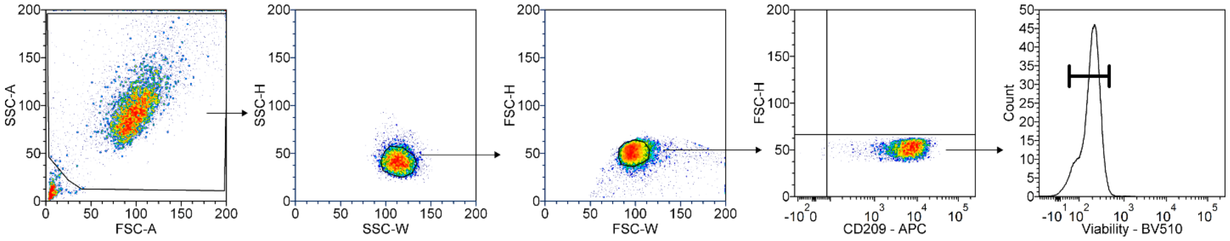

Fig. S1 Gating strategy used to identify human moDCs. Doublets were excluded by consecutive gating of FSC-H/FSC-W and SSC-H/SSC-W. MoDCs were selected based on the expression of the specific moDC marker DC-SIGN (CD209). Dead cells were excluded by using the Zombie Aqua™ Fixable Viability Kit.

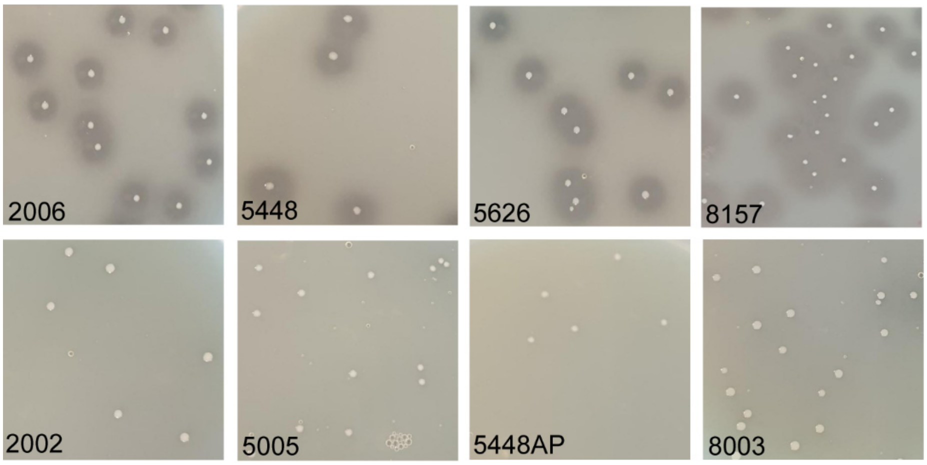

Fig. S2 CovR/S TCS functionality as assessed via SpeB proteolytic activity on casein agar plates. Presence of hydrolysis zones around the colonies was categorized as CovR/S$^+$. Lack of hydrolysis zones was categorized as CovR/S$^-$.

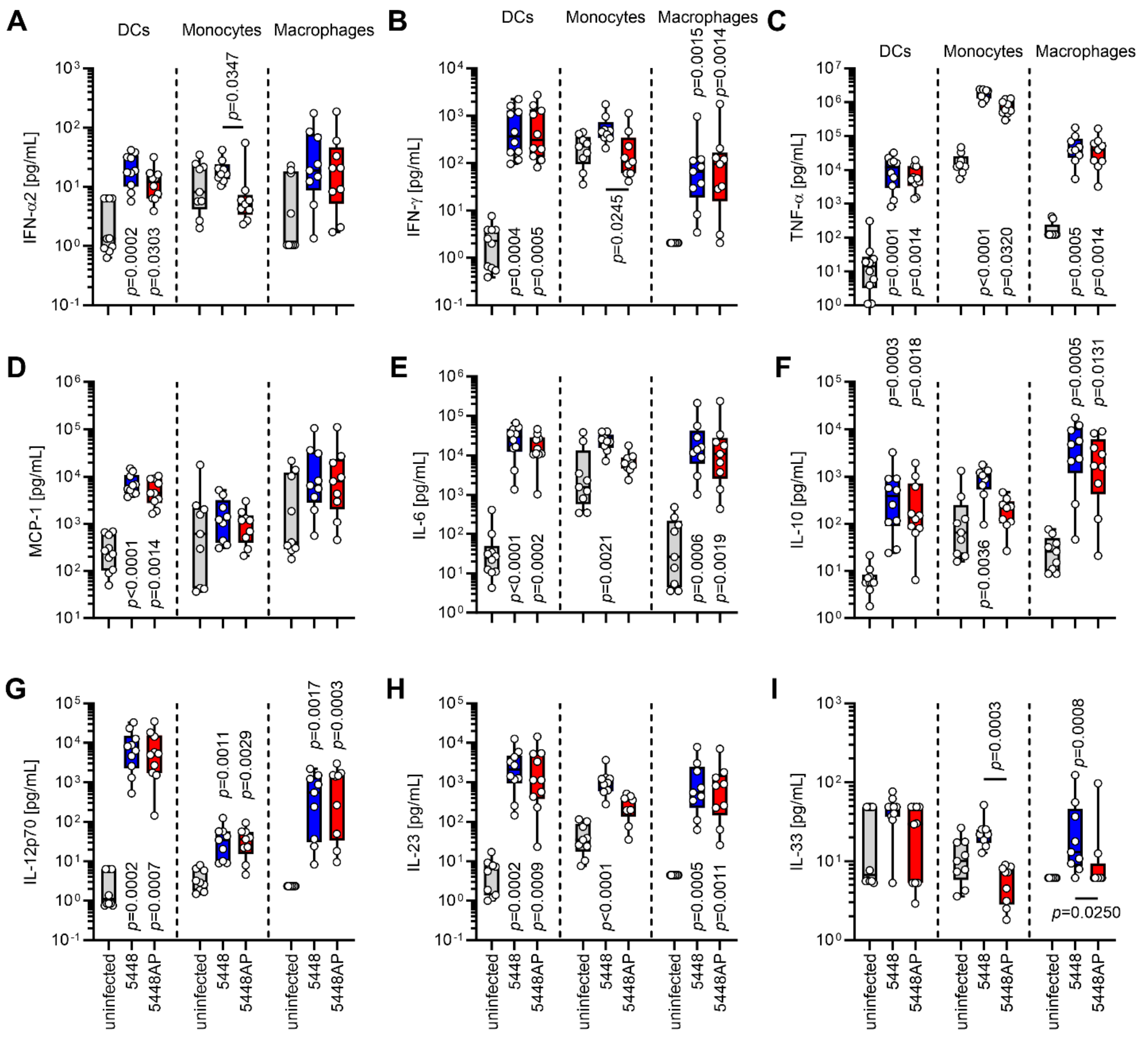

Fig. S3 MoDCs, monocytes, or monocyte-derived macrophages were infected with 5448 or 5448AP and cytokine secretion by myeloid cells was measured via a multiplex assay (n≥8). Displayed are concentrations of IFN-α2 (A), IFN-γ (B), TNF-α (C), MCP-1 (D), IL-6 (E), IL-10 (F), IL-12p70 (G), IL-23 (H), and IL-33 (I) in supernatants of (un)infected cells. The data are displayed as box plots. Each dot represents one independent experiment with cells from one donor (n≥8). The level of significance was determined using Kruskal-Wallis test with Dunn's posttest.

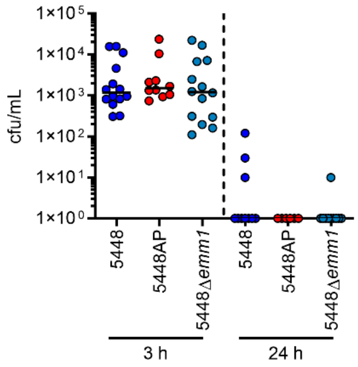

Fig. S4 MoDCs eliminate intracellular GAS. Viable intracellular bacteria were determined at indicated time points post infections. Each dot represents one independent experiment with cells from one donor (n≥10). Horizontal lines denote median value.

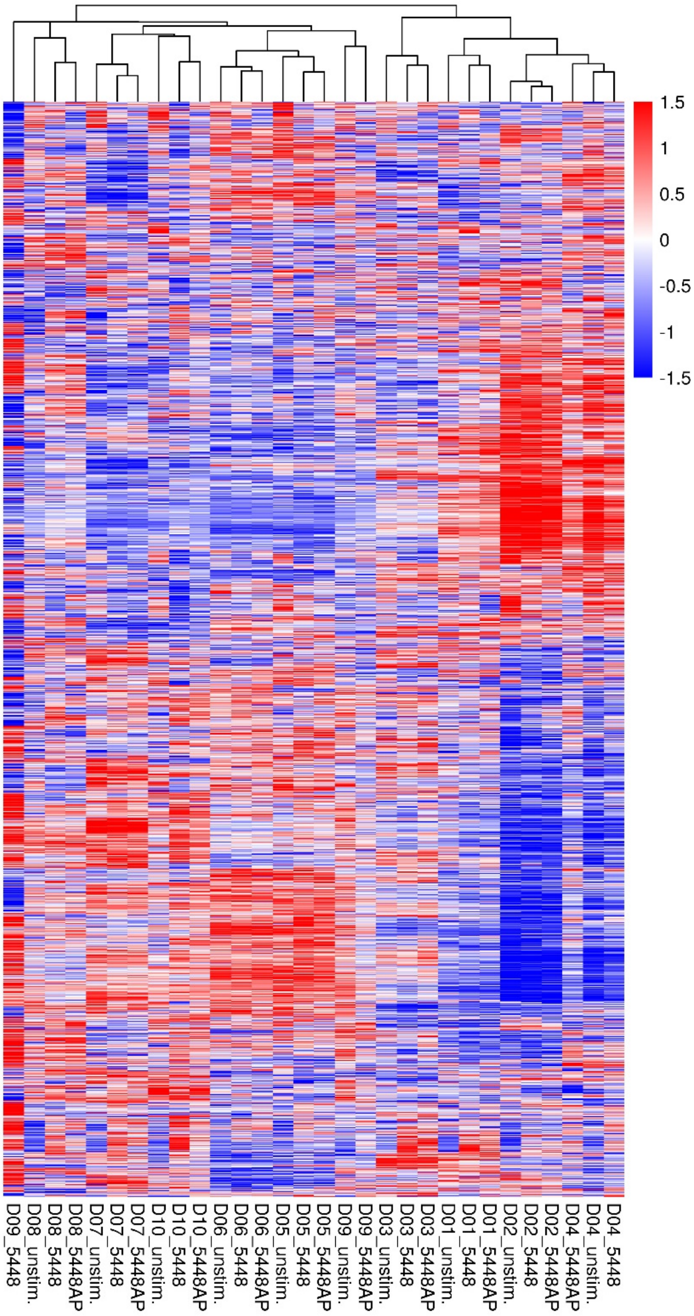

Fig. S5 Heatmap (z-scored) depicting protein intensities (maxLFQ) in moDCs 6 h post infection. Each column represents one condition for one respective donor (n=10). In total, 5857 protein groups were identified. For quantification 4801 protein groups, identified with two or more peptides and found in 50% of the individual condition replicates, were used.

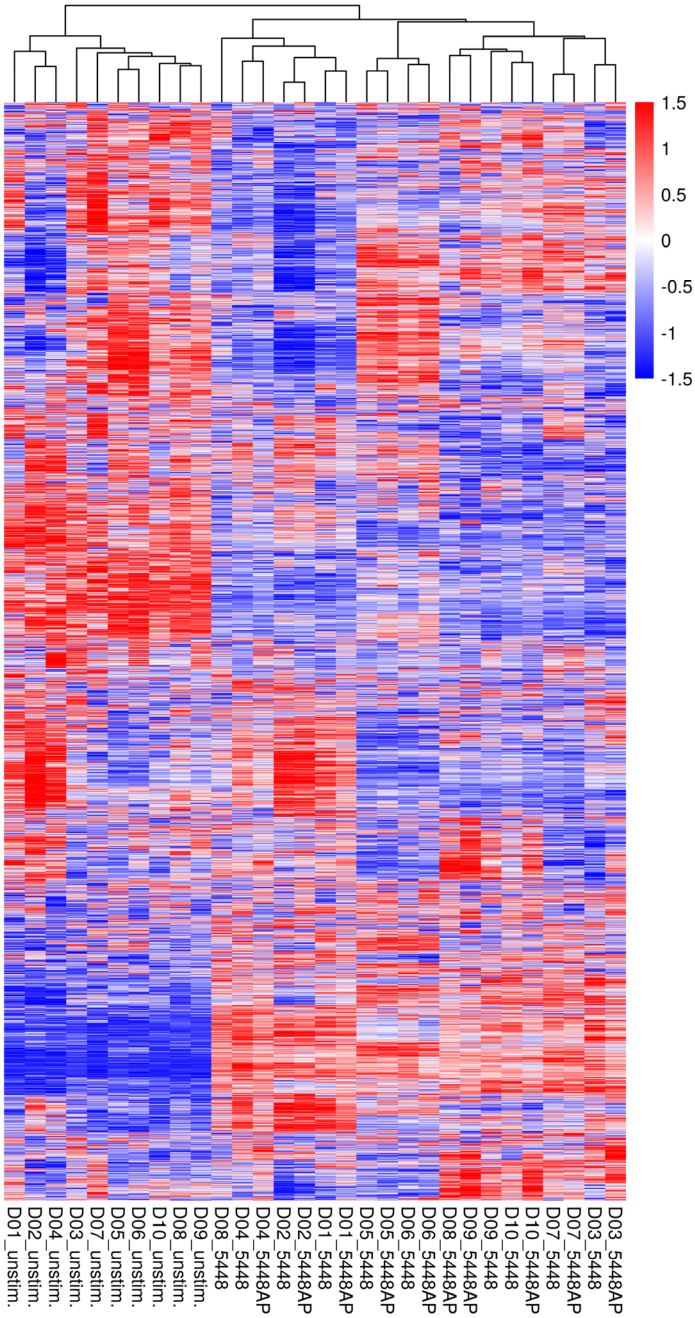

Fig. S6 Heatmap (z-scored) depicting protein intensities (maxLFQ) in moDCs 24 h post infection. Each column represents one condition for one respective donor (n=10). In total, 5857 protein groups were identified. For quantification 4801 protein groups, identified with two or more peptides and found in 50% of the individual condition replicates, were used.

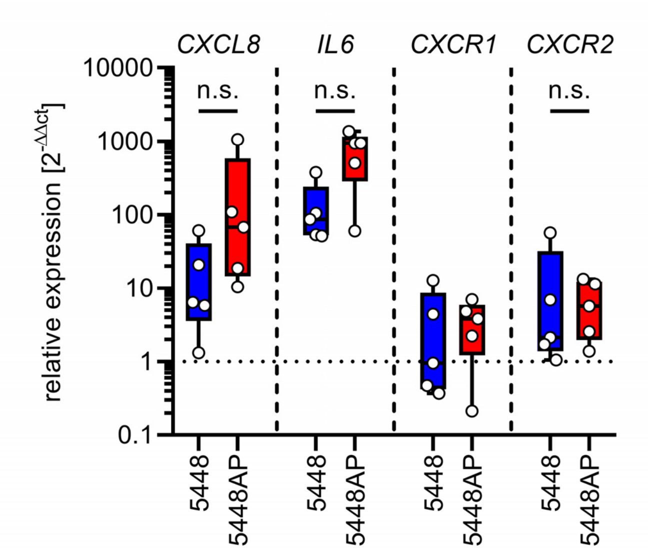

Fig. S7 Gene expression analyses in infected moDCs. Human primary moDCs were infected with GAS 5448 or 5448AP (MOI 10). Extracellular bacteria were killed by substituting the media with antibiotics. Gene expression of genes encoding for CXCL8, IL-6, CXCR1 and CXCR2 relative to uninfected controls (dashed line) are shown. The data are displayed as box plots. Each dot represents one independent experiment with cells from one donor (n=5).

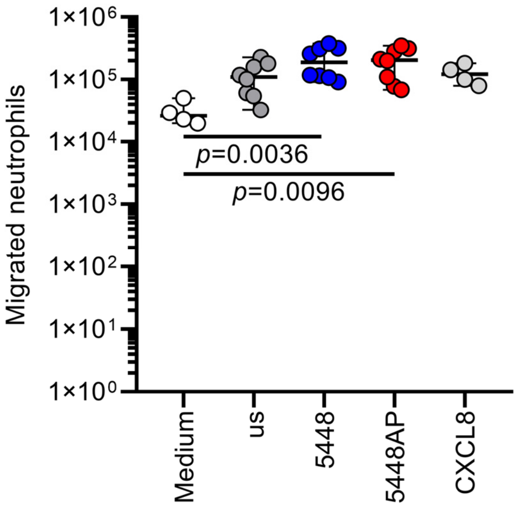

Fig. S8 Chemotactic effects of infection supernatants derived from 5448/5448AP infected moDCs on primary neutrophils. Neutrophil migration was determined using a transwell assay system. Neutrophils were left to migrate against supernatants of (un)infected moDCs or 12.5 ng×mL^{-1} IL-8 for 2 h. Migration of neutrophils from four donors (n=4) was measured against conditioned culture medium derived from (un)infected moDCs from 2 individual donors. RPMI media was used as a medium control to assess spontaneous migration of neutrophils.

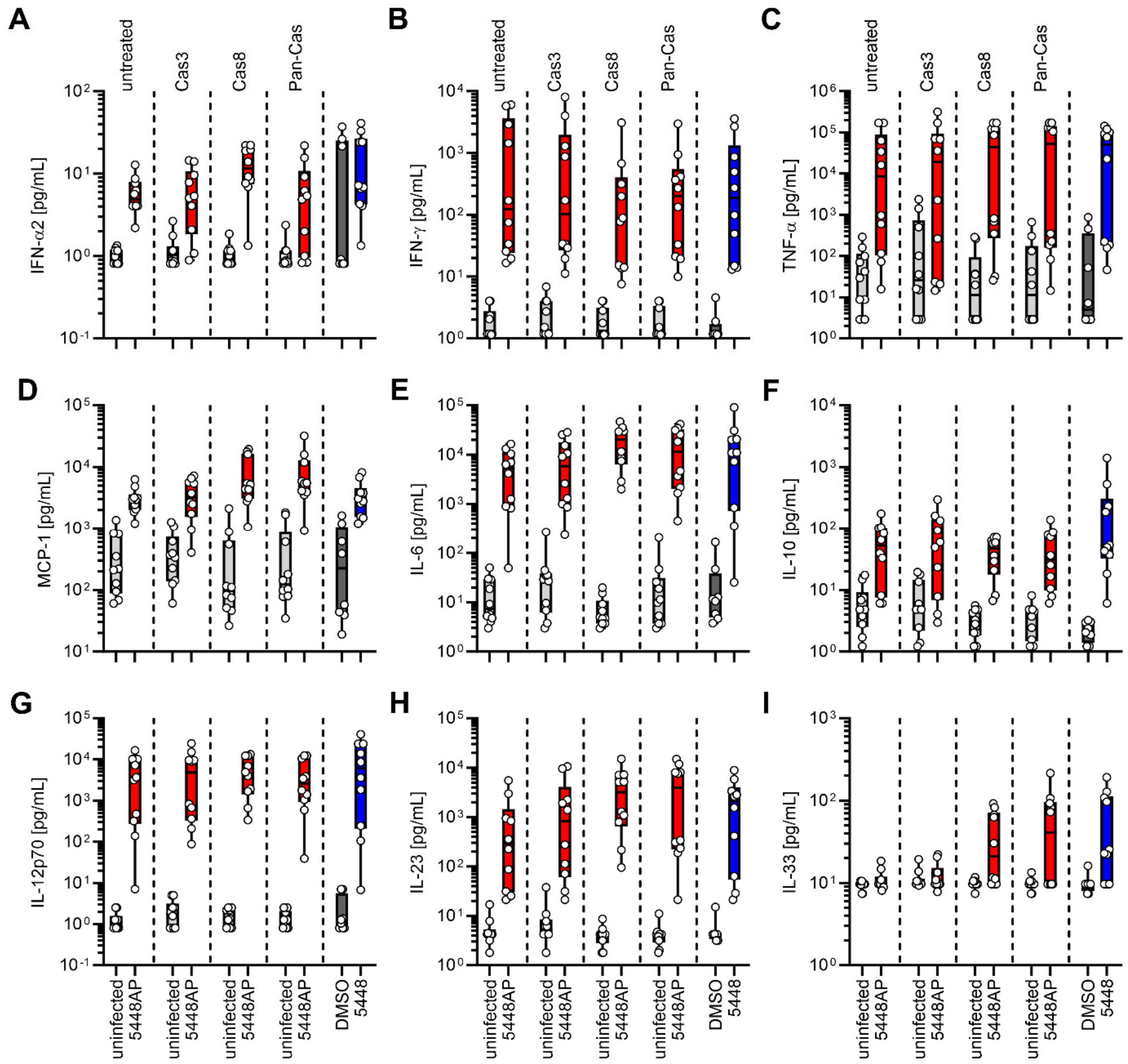

Fig. S9 MoDCs were treated with caspase inhibitors (caspase-3: Cas3/7-Inhibitor I, Ac-DEVD-cho; caspase-8: z-IETD-fmk, pan-capase: z-VAD-fmk) and subsequently infected with 5448AP. Cytokine secretion by moDCs was measured via a multiplex assay. The concentrations of IFN-α2 (A), IFN-γ (B), TNF-α (C), MCP-1 (D), IL-6 (E), IL-10 (F), IL-12p70 (G), IL-23 (H), and IL-33 (I) were measured in supernatants of (un)infected moDCs. The data are displayed as box plots. Each dot represents one independent experiment with cells from one donor (n=10). A separate uninfected control with the respective inhibitor treatment was performed for each 5448AP infection. Untreated: indicates 5448AP infection without inhibitors. Each infection was compared to its respective uninfected controls. The level of significance was determined using Mann-Whitney U-test. Exact *p*-values are displayed in Table S1.

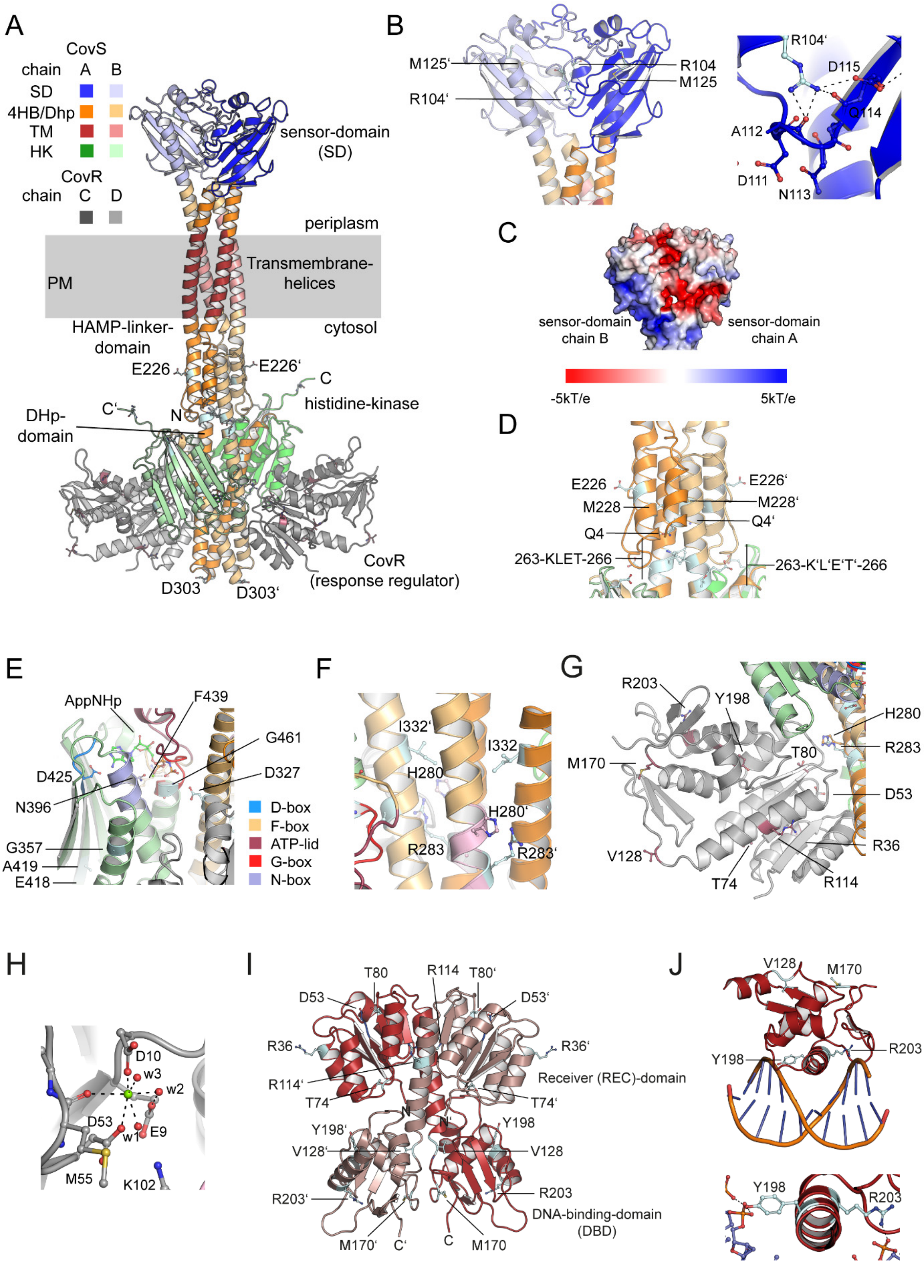
A
CovS
chain A B
SD
4HB/Dhp
TM
HK
CovR
chain C D
sensor-domain (SD)
periplasm
PM
Transmembrane-helices
HAMP-linker-domain
cytosol
E226
E226'
C
C'
N
histidine-kinase
DHp-domain
CovR (response regulator)
D303
D303'
B
M125'
R104'
R104
M125
R104'
D115
Q114
A112
D111
N113
C
sensor-domain chain B
sensor-domain chain A
-5kT/e
5kT/e
D
E226
E226'
M228
M228'
Q4
Q4'
263-KLET-266
263-K'L'E'T'-266
E
AppNHp
F439
G461
D425
D327
N396
G357
A419
E418
D-box
F-box
ATP-lid
G-box
N-box
F
I332'
I332
H280
H280'
R283
R283'
G
R203
Y198
H280
R283
M170
T80
D53
V128
R36
T74
R114
H
D10
w3
w2
D53
w1
E9
M55
K102
I
T80
R114
T80'
D53
D53'
R36
R36'
R114'
Receiver (REC)-domain
T74
T74'
N
N'
Y198'
Y198
V128'
V128
R203'
R203
DNA-binding-domain (DBD)
M170'
C'
C
M170
J
V128
M170
Y198
R203
Y198
R203

Fig. S10: Structural analyses of the overall fold of the CovS/CovR histidine kinase response regulator complex by AlphaFold2.[5,6]

(A) AlphaFold2 was used to predict the overall structure of the tetrameric $CovR_2$•2CovS complex. As described for two-component signaling systems, the histidine kinase CovS dimerizes. It consists of a periplasmic sensor-domain (SD), a section that contains the transmembrane helices, a HAMP (histidine kinases, adenylyl cyclases, methyl-accepting chemotaxis proteins and phosphatases)-linker-domain forming a four-helix bundle, a dimerization and histidine phosphotransfer (DHp)-domain containing the phosphorylated histidine residue (H280) and the histidine-kinase (HK)-domain containing the ATP-binding site. The response regulator CovR binds to the DHp-domain and HK-domain. Two molecules of CovS bind to a CovR-dimer. CovR is phosphorylated at an aspartate residue, D53, that induces dissociation from CovS to bind to the DNA. The structure shows that CovS is in a state incompatible with phosphorylation of H280 and transfer of the phosphoryl-group to D53, suggesting that activation of the sensor-domain by ligand binding, such as LL-37, or by a pH shift into the acidic range. The figure was created by PyMOL.[8]

(B) The sensor domain (SD) of the histidine kinase CovS dimerizes by formation of interactions of residues from chain A with residues of chain B. M125 and R104 were found to be mutated to isoleucine and histidine, respectively. Mutation of R104H would interfere with formation of several interactions. R104 from chain B/A forms an important salt bridge with the side chain of D115 of chain A/B, i.e. *in trans*, thereby interconnecting the dimer. Moreover, R104 of chain B/A forms hydrogen bonds to the main chain carbonyl of A112 and D111 of chain A/B, respectively. Asp115 is part of a large electrostatic network containing several acidic residues (right panel). The side chain of Asp115 shows an increased pK_a value of 6.09 for chain A and of 5.85 for chain B, as determined by the APBS-PDB2PQR software suite.[7] This would allow alteration of the protonation state dependent of the pH and thereby to translate changes in the pH-value into altered conformations. It is postulated that CovS senses acidic shifts of the environmental pH to approximately pH 5.5. Upon protonation of the Asp115 side chains of chainA/B, formation of the salt bridge with R104 in chain B/A is abolished. This might result in alteration of the sensor-domain conformation that is further propagated into the cell, finally activating the histidine kinase activity. The impact of the mutation of M125I is less obvious. It might interfere with the conformation of the SD. The pK_a-values were determined by the APBS-PDB2PQR software suite.[7] The figure was created by PyMOL.[8]

(C) The surface of the sensor-domain contains a strong acidic patch. The electrostatic surface potential is shown from -5kT/e (negatively charged, red) to +5kT/e (positively charged, blue). D115 of chain A is part of this acidic patch visible in this representation. The acidic patch of chain B is on the opposite surface area. The figure was created by the APBS plugin in PyMOL.[8]

(D) Residues that are mutated in CovS cluster at the transition from the HAMP-domain to the DHp-domain. The residues E226, M228, and Q4 were found to be mutated, and patch 163-KLET-266 was found to be deleted. This area is important to translate the extracellular activation of the sensor-domain (SD) into the activation of the histidine kinase (HK). To this end, a conformational change might be elicited that results in the rotation of the [illegible]-helices of the HAMP- and DHp-domains that bring H280 located in DHp-domain in proximity to the ATP-bound to the HK-domain. Mutation of residues in this area might impair the signal-transduction from the sensor-domain to finally activate the histidine kinase. The figure was created by PyMOL.[8]

(E) Structural organization of the histidine kinase domain. The CovS HK-domain shows all sequence motifs essential for an active histidine kinase. The D-box contains an aspartate, i.e. D425, that directly contacts the exocyclic amino group of the adenine base at C6, thereby creating specificity for adenine nucleotides. The F-box as a central element contains F438. The ATP-lid covers the ATP-molecule. The G-box contains several glycine side chains. This sequence element contacts the [illegible]- and [illegible]-phosphates and is similar to a P-loop described for other nucleotide binding proteins. It is needed for ATP-binding and forms an oxyanion hole to stabilize a negative charge occurring at the phosphate oxygen during catalysis. G461 within the G-box was found to be mutated to serine. This mutation might directly interfere with ATP/ADP-binding and might impair phosphoryl-group transfer. The stretch from E418 to K432 was found to be deleted. This will render the kinase inactive, as ATP/ADP-binding will be impaired and the HK-fold will be disrupted. Finally, the N-box contains a conserved asparagine, i.e. N396. N396 is involved in coordinating the bound Mg^{2+}-ion needed for nucleotide binding and to allow the nucleophilic attack of the activated H280 for phosphoryl-group transfer. Mg^{2+}-ATP was modelled by superimposing the CovS•CovR structure and the structure 4KP4 by PyMOL.[8] K399 of the N-box forms a salt bridge with D327 of the DHp-domain, thereby positioning the HK-domain in the conformation analyzed here. D327 within the DHp domain was found to be mutated to asparagine. Related histidine kinases have a glutamine at the analogous position. As asparagine and glutamine have similar physicochemical properties but are sterically different, an impact of CovS•CovR function cannot be excluded. Another mutation identified in the HK-domain is K498N at the C-terminus. This residue is solvent-exposed and might not directly affect CovS activity. K is a positively charged residue at physiological pH and might be important for solubility of CovS. Furthermore, lysines were known to be post-translationally modified, i.e. by lysine acetylation. Mutation of K498N would abolish this. The figure was created by PyMOL.[8].

(F) Closeup of the CovS DHp-domain showing position of I332 and the phosphoryl-group acceptor H280 and R283. I332 was found to be mutated to valine. Although this is an exchange of residues with similar physicochemical properties, both being hydrophobic

side chains, they are sterically different. As predicted by marcoil I332 is positioned at position "a" of the coiled-coil heptad repeat [2]. This means that it is directly involved in the formation of the interface of the coiled-coil structure. Exchange of I332 to V, therefore, might affect the coiled-coil structure. As a direct consequence this could affect the position of the phosphoryl-group acceptor H280 and E281 serving as hydrogen bond acceptor orienting and activating H280 for nucleophilic attack on the □-phosphate of the ATP molecule. Notably, H280 shows a strongly reduced pK_a value of 2.09 (chain A)/2.10 (Chain B) compared to the free amino acid. This shows that H280 is deprotonated in this structural environment and able to act as a strong nucleophile during catalysis. R283 is postulated to be important for phosphoryl-group transfer by contacting the negatively-charged ATP-phosphoryl groups. Similar to an arginine-finger in small GTP-binding proteins, this might improve the nucleophilic attack of H280 on the □-phosphate by neutralizing the negative charge emerging in the transition state of catalysis. Besides, R383 contacts H280. Deletion of R283 affects the pK_a of H280, shifting it to 5.24 (chain A)/5.18 (chain B). This shows that deletion of R283 decreases nucleophilicity of H280. R283 was found to be deleted in CovS variants. While AlphaFold2 structure predictions (not shown) suggest that deletion of R283 does not affect CovS structure, it will impair catalytic activity of HK. The pK_a-values were determined by the APBS-PDB2PQR software suite.[7] The figure was created by PyMOL.[8]

(G) Structure of the response-regulator CovR bound to histidine kinase CovS. Residues found to be mutated in CovR are shown in dark red. R36 and V128 are positioned towards the solvent. It was found that R36 is mutated to cysteine. Mutation of a solvent exposed residue to cysteine might affect protein function by formation of cysteine disulfide bonds, if occurring under non-reducing conditions. M170 is solvent exposed but might also play a structural role. i.e. as hydrophobic amino acid and by acting hydrogen bond acceptor via bridging water molecules not visible in this structural model. T80 was found to be mutated to alanine. T80 is located not too far away from D53, the phosphoryl-group acceptor site in CovR. The hydroxyl-group at the side chain of T80 might play a structural role, i.e. via formation of hydrogen bonds with bridging water molecule. This cannot be achieved by an alanine side chain. If this manifests in CovS•CovR activity needs to be evaluated. R203 and Y198 are located within the CovR DNA-binding helix. In the CovS-bound state R203 is solvent exposed and Y198 might play a structural role via formation of hydrogen bonds between the side chain hydroxyl and bridging water molecules. The figure was created by PyMOL.[8]

(H) Superposition of the AlphaFold2 CovR•CovS structure with the structure of the response regulator CheY (PDB: 2CHE) suggests that the response-regulator CovR binds Mg^{2+}. The Mg^{2+}-binding site is totally conserved. The Mg^{2+} is hexacoordinated by three water molecules (w1, w2, w3), the side chain of D110, the main chain carbonyl oxygen of M55 (N59 in CheY) and by the side chain of the phosphoryl-group acceptor D53. E9 positions w2 by formation of a hydrogen bond. K102 contacts D53 by formation of a salt bridge. During catalysis the carboxylate of D53 would act as a nucleophile and performs an in-line attack to the phosphoimidazol ring of H280 with H280 acting as a leaving group. It is suggested that the bound Mg^{2+} stabilizes the pentavalent bipyramidal transition state during phosphoryl-transfer reactions. In analogy to CheY, K102 in CovR might be dispensable for the phosphoryl-transfer reaction. However, it might bind to the phosphorylated D53 carboxylate thereby eliciting conformational changes. This might result in dissociation of CovR from CovS adopting a conformation that allows CovR to bind to the target DNA sequence. The figure was created by PyMOL[8].

(I) Overall conformation of the non-complexed, non-phosphorylated CovR dimer as predicted by AlphaFold2. CovR can be separated in a C-terminal DNA-binding domain (aa129-227) with the DNA-binding helix containing R203 and Y198 and a N-terminal receiver (REC)-domain (4-117) that contains D53 that is phosphorylated by the histidine kinase of CovS in order to act as a transcriptional regulator. The conformation shown here is not compatible with DNA-binding at both DNA-binding sites. The figure was created by PyMOL.[8]

(J) Closeup of the DNA-binding site of CovR in complex with DNA. CovR was superimposed with 1GXP. CovR inserts an □-helix into the major groove of the DNA. R203 and Y198 form contacts to the sugar-phosphate backbone. The side chain hydroxyl of Y198 forms a hydrogen bond with the phosphate of the DNA-backbone, R203 forms a salt bridge with the phosphate of the DNA-backbone. In this conformation, both residues would contribute to DNA-binding affinity but not to create sequence specificity. However, binding to DNA might alter the conformation and a crystal structure of CovS in complex with dsDNA would be needed to judge this. R203 was shown to be mutated to serine, which is not compatible with creating the interactions with DNA. Y198 was found to be deleted in CovR variants. Mutation of R203S will likely decrease binding affinity while possibly retaining some DNA-binding. However, deletion of Y198 will most likely result in variant not capable to bind DNA as all residues that mediate DNA-binding, i.e. R200, R203 and K205, are located C-terminally of Y198 and will not be properly oriented to allow DNA-binding. The figure was created by PyMOL[8]

Table S1 qRT-PCR primer used in this study

Target	Sequence (5'-3')
CXCL8 forward	GAGCACTCCATAAGGCACAAA
CXCL8 reverse	ATGGTTCCTTCCGGTGGT
IL6 forward	AATTCGGATCATCCTCGACGG
IL6 reverse	GGTTGTTTTCTGCCAGTGCCT
CXCR1 forward	TTTGTTTGTCTTGGCTGCTG
CXCR1 reverse	AGTGTACGCAGGGTGAATCC
CXCR2 forward	ACAGCTACTTGGGAGGCTGA
CXCR2 reverse	TGCAGTGGTCACACCATTTT
β_actin forward	CTCTTCCAGCCTTCCTTCCT
β_actin reverse	AGCACTGTGTTGGCGTACAG

Table S3 Literature review on previously characterized *covR/S* mutations

**Amino acid substitutions**	**CovR/S phenotype**	**Reference**
I332V in CovS	functional	[3]
G461S in CovS	dysfunctional	[4]
E226G in CovS	dysfunctional	[4]
R203S in CovR	dysfunctional	[1]
M1I in CovS	dysfunctional	[10]
T214P in CovS	dysfunctional	[10]
truncation from aa300 in CovS	dysfunctional	[12]
truncation from aa202 in CovS	dysfunctional	[11]

Table S4. Parameters for mass spectrometry

reversed phase liquid chromatography (RPLC)

instrument	Ultimate 3000 RSLC (Thermo Scientific)
trap column	75 µm inner diameter, packed with 3 µm C18 particles (Acclaim PepMap100, Thermo Scientific)
analytical column	Accucore 150-C18, (Thermo Fisher Scientific)<br>25 cm x 75 µm, 2,6 µm C18 particles, 150 Å pore size
buffer system	binary buffer system consisting of 0.1% acetic acid in HPLC-grade water (solvent A) and 100% ACN in 0.1% acetic acid (solvent B)
flow rate	300 nl/min
gradient	(0min-2% B<br>2min-5% B<br>10min-7% B<br>70min-25% B<br>75min-40% B<br>77min-90% B<br>83min-90% B<br>85min-2% B<br>95min-2%B
column oven temperature	40°C
Mass spectrometry	
instrument	Orbitrap Exploris 480
electrospray	Nanospray Flex™ Ion Source
operation mode	data-independent
Full Scan Properties	
MS scan resolution	120000
AGC target	3e6 (300%)
maximum ion injection time for the MS scan	60 ms
Scan range	350 to 1200 m/z
Microscans	1
Polarity	positive
RF Lens	50%
Spectra data type	profile
Dia Properties (MS2)	
Resolution	30,000

maximum ion injection time for the MS/MS scans	auto
Normalized AGC target	3E6
Spectra data type	profile
Microscans	1
isolation window	66
Isolation window width	13 m/z
Window overlay	2 m/z
Fixed first mass	200
HCD collision energy	30%

Table S6 Exact *p*-values of statistical analysis of cytokine secretion by moDCs after caspase-inhibition (n=10). A separate uninfected control with the respective inhibitor treatment was performed for each 5448AP infection. The level of significance was determined using Mann-Whitney *U*-test.

	**untreated vs. 5448AP**	**Cas3 Inh vs. 5448AP**	**Cas8 Inh vs. 5448AP**	**PanCas Inh vs. 5448AP**	**untreated vs. 5448**
**IL-1β**	$p=0.0045$	$p=0.0385$	$p<0.0001$	$p=0.0055$	$p=0.0045$
**IFNα**	$p<0.0001$	$p=0.0019$	$p<0.0001$	$p=0.0094$	$p<0.0001$
**IFNγ**	$p<0.0001$	$p<0.0001$	$p<0.0001$	$p<0.0001$	$p<0.0001$
**TNF**	$p=0.0020$	$p=0.0286$	$p=0.0007$	$p=0.0036$	$p=0.0007$
**MCP-1**	$p<0.0001$	$p=0.0002$	$p<0.0001$	$p<0.0001$	$p<0.0001$
**IL-6**	$p<0.0001$	$p<0.0001$	$p<0.0001$	$p<0.0001$	$p<0.0001$
**IL-8**	$p=0.0068$	$p=0.0115$	$p=0.0068$	n.s.	$p=0.0003$
**IL-10**	$p=0.0014$	$p=0.0109$	$p<0.0001$	$p<0.0001$	$p=0.0321$
**IL-12p70**	$p<0.0001$	$p<0.0001$	$p<0.0001$	$p<0.0001$	$p<0.0001$
**IL-18**	n.s.	n.s.	$p=0.0014$	$p=0.0084$	$p=0.0021$
**IL-23**	$p<0.0001$	$p<0.0001$	$p<0.0001$	$p<0.0001$	$p<0.0001$
**IL-33**	n.s.	n.s.	$p=0.0159$	n.s.	$p=0.0027$

Table legends for Additional File 2.

Table S2 CovR/S phenotype of GAS isolates recovered from the INFECT cohort. Type of infection (poly-/monomicrobial), involvement of septic shock, CovR/CovS mutations as identified by bioinformatics, and CovR/S functionality. Data was published in [9] and re-analyzed based on CovR/S functionality. CovR/S functionality was assessed by literature review (Table S2) and protein structure prediction (Fig. S8). Yellow color indicates dysfunctional CovR/S system.

Table S5 Statistical analyses of the abundance patterns of proteins of (un)infected moDCs 6 h and 24 h post indicated infections. Color code: blue: log_2 fold change below 0; red: log_2 fold change above 0.

References

1. Dalton T.L. and Scott J.R. CovS Inactivates CovR and Is Required for Growth under Conditions of General Stress in <i>Streptococcus pyogenes</i>. Journal of Bacteriology 186(12):3928-3937, 2004.
2. Delorenzi M. and Speed T. An HMM model for coiled-coil domains and a comparison with PSSM-based predictions. Bioinformatics 18(4):617-625, 2002.
3. Horstmann N., Sahasrabhojane P., Saldaña M., Ajami N.J., Flores A.R., Sumby P., Liu C.-G., Yao H., Su X., Thompson E. and Shelburne S.A. Characterization of the Effect of the Histidine Kinase CovS on Response Regulator Phosphorylation in Group A Streptococcus. Infection and Immunity 83(3):1068-1077, 2015.
4. Ikebe T., Ato M., Matsumura T., Hasegawa H., Sata T., Kobayashi K. and Watanabe H. Highly Frequent Mutations in Negative Regulators of Multiple Virulence Genes in Group A Streptococcal Toxic Shock Syndrome Isolates. PLOS Pathogens 6(4):e1000832, 2010.
5. Jumper J., Evans R., Pritzel A., Green T., Figurnov M., Ronneberger O., Tunyasuvunakool K., Bates R., Zidek A., Potapenko A., Bridgland A., Meyer C., Kohl S.A.A., Ballard A.J., Cowie A., Romera-Paredes B., Nikolov S., Jain R., Adler J., Back T., Petersen S., Reiman D., Clancy E., Zielinski M., Steinegger M., Pacholska M., Berghammer T., Bodenstein S., Silver D., Vinyals O., Senior A.W., Kavukcuoglu K., Kohli P. and Hassabis D. Highly accurate protein structure prediction with AlphaFold. Nature 596(7873):583-589, 2021.
6. Jumper J. and Hassabis D. Protein structure predictions to atomic accuracy with AlphaFold. Nat Methods 19(1):11-12, 2022.
7. Jurrus E., Engel D., Star K., Monson K., Brandi J., Felberg L.E., Brookes D.H., Wilson L., Chen J., Liles K., Chun M., Li P., Gohara D.W., Dolinsky T., Konecny R., Koes D.R., Nielsen J.E., Head-Gordon T., Geng W., Krasny R., Wei G.W., Holst M.J., McCammon J.A. and Baker N.A. Improvements to the APBS biomolecular solvation software suite. Protein Sci 27(1):112-128, 2018.
8. Madsen M.B., Skrede S., Perner A., Arnell P., Nekludov M., Bruun T., Karlsson Y., Hansen M.B., Polzik P., Hedetoft M., Rosén A., Saccenti E., Bergey F., Martins dos Santos V.A.P., Bidstrup D., Bærnthsen N.F., Frendø G.H., Jansen E.C., Madsen L.B., Müller R.B., Pedersen E.M.J., Petersen M.W., Ravn F., Smidt-Nielsen I.F.G., Wahl A.M., Wulffeld S., Aronsson S., Rosemar A., Trogen J., Nedrebø T., Oppegaard O., Rath E., Sævik M., Norrby-Teglund A., Hyldegaard O. and group I.s. Patient’s characteristics and outcomes in necrotising soft-tissue infections: results from a Scandinavian, multicentre, prospective cohort study. Intensive Care Medicine 45(9):1241-1251, 2019.
9. Palma Medina L.M., Rath E., Jahagirdar S., Bruun T., Madsen M.B., Stralin K., Unge C., Hansen M.B., Arnell P., Nekludov M., Hyldegaard O., Lourda M., Santos V., Saccenti E., Skrede S., Svensson M. and Norrby-Teglund A. Discriminatory plasma biomarkers predict specific clinical phenotypes of necrotizing soft-tissue infections. J Clin Invest 131(14), 2021.
10. Shumba P., Sura T., Moll K., Chakrakodi B., Tolken L.A., Hossmann J., Hoff K.J., Hyldegaard O., Nekludov M., Svensson M., Arnell P., Skrede S., Group I.S., Norrby-Teglund A. and Siemens N. Neutrophil-derived reactive agents induce a transient SpeB negative phenotype in Streptococcus pyogenes. J Biomed Sci 30(1):52, 2023.
11. Sumby P., Whitney A.R., Graviss E.A., DeLeo F.R. and Musser J.M. Genome-Wide Analysis of Group A Streptococci Reveals a Mutation That Modulates Global Phenotype and Disease Specificity. PLOS Pathogens 2(1):e5, 2006.
12. Walker M.J., Hollands A., Sanderson-Smith M.L., Cole J.N., Kirk J.K., Henningham A., McArthur J.D., Dinkla K., Aziz R.K., Kansal R.G., Simpson A.J., Buchanan J.T., Chhatwal G.S., Kotb M. and Nizet V. DNase Sda1 provides selection pressure for a switch to invasive group A streptococcal infection. Nature Medicine 13(8):981-985, 2007.
